# Phenotypic variance explained by local ancestry in admixed African Americans

**DOI:** 10.3389/fgene.2015.00324

**Published:** 2015-10-29

**Authors:** Daniel Shriner, Amy R. Bentley, Ayo P. Doumatey, Guanjie Chen, Jie Zhou, Adebowale Adeyemo, Charles N. Rotimi

**Affiliations:** Center for Research on Genomics and Global Health, National Human Genome Research InstituteBethesda, MD, USA

**Keywords:** ancestry, complex traits, health disparities, phenotypic variance explained, random effects

## Abstract

We surveyed 26 quantitative traits and disease outcomes to understand the proportion of phenotypic variance explained by local ancestry in admixed African Americans. After inferring local ancestry as the number of African-ancestry chromosomes at hundreds of thousands of genotyped loci across all autosomes, we used a linear mixed effects model to estimate the variance explained by local ancestry in two large independent samples of unrelated African Americans. We found that local ancestry at major and polygenic effect genes can explain up to 20 and 8% of phenotypic variance, respectively. These findings provide evidence that most but not all additive genetic variance is explained by genetic markers undifferentiated by ancestry. These results also inform the proportion of health disparities due to genetic risk factors and the magnitude of error in association studies not controlling for local ancestry.

## Introduction

Two statistical techniques to map disease risk variants are widely used with genome-wide genetic data, association testing and admixture mapping. Genetic association refers to a correlation of phenotype and genotype. In association studies, population structure can be a confounder, leading to both false positive and false negative associations. Population structure is typically described by two models, population stratification and admixture. Population stratification refers to systematic differences in allele frequencies between subgroups of the sample, also called strata. Each individual belongs to one stratum. Methods to identify and control for population stratification include genomic control (Devlin and Roeder, 1999), structured association testing (Pritchard et al., 2000), principal components analysis (Price et al., 2006), and linear mixed models (Eu-Ahsunthornwattana et al., 2014).

Linear mixed models account for relatedness by making use of pairwise genetic similarity. The kinship coefficient is a measure of the expected genetic similarity between two individuals, usually defined as the expected probability that two alleles, one sampled at random from each individual, are identical by descent. The realized genetic similarity between a pair of individuals varies because of segregation and also varies by locus (Hayes et al., 2009). The genetic similarity matrix can be estimated from a genome-wide sample of single nucleotide polymorphisms (SNPs) and can be used to estimate the proportion of phenotypic variance explained by additive genetic variance (Yang et al., 2011a).

Admixture refers to mating between two or more previously isolated populations. An admixed individual's genome is a mosaic of chromosomal segments with ancestry variable by locus. The ancestral population of origin at each locus for each admixed individual may be probabilistically identifiable. Characterization of ancestry for an admixed individual involves ancestral proportions measured as genome-wide averages, known as global ancestry, and ancestral states inferred for each individual at each locus, known as local ancestry (Padhukasahasram, 2014). Admixture mapping is designed specifically to test the correlation of phenotype and local ancestry (Winkler et al., 2010).

When working with genotype data, controlling for effects of global ancestry can be achieved by including individual admixture proportions. However, control of global ancestry does not control local ancestry, nor does control of local ancestry control global ancestry (Qin et al., 2010; Shriner et al., 2011a). Consequently, in admixed individuals, estimates of the proportion of phenotypic variance explained by genotype are confounded by local ancestry.

The ancestral similarity matrix is a construct for use with samples of individuals from admixed populations, such as African Americans (Zaitlen et al., 2014). The ancestral similarity matrix can be estimated from local ancestry inferred from a genome-wide sample of SNPs and can be used to estimate the proportion of phenotypic variance explained by additive genetic variance (Zaitlen et al., 2014). Here, we extend this approach to investigate the proportion of phenotypic variance explained by local ancestry in two epidemiological studies of admixed African Americans. We show that the proportion of phenotypic variance explained by local ancestry can be interpreted in several ways: (1) it provides an upper bound on how much phenotypic variance is accessible to admixture mapping, (2) it quantifies the magnitude of confounding in association studies of genotype by local ancestry remaining even after adjustment for global ancestry, and (3) it informs health disparities research by directly estimating ancestry effects on outcomes.

## Materials and methods

### Study descriptions

The Howard University Family Study (HUFS) is a population-based observational study of African Americans from Washington, D.C. Ethical approval was obtained from the Howard University Institutional Review Board. All subjects gave written informed consent in accordance with the Declaration of Helsinki. Data are available upon collaboration with Dr. Charles N. Rotimi. HUFS comprised 1976 individuals, 1055 of whom were unrelated (Adeyemo et al., 2009). Genotyping was performed using the Affymetrix Genome-Wide Human SNP Array 6.0, with quality control as described previously (Adeyemo et al., 2009; Shriner et al., 2009). Also as described previously (Shriner et al., 2012), local ancestry estimates (0, 1, or 2 chromosomes of African ancestry) were obtained for 797, 831 autosomal SNPs using LAMPANC version 2.3 (Sankararaman et al., 2008) and HapMap Phase II+III CEU and YRI reference allele frequencies (http://hapmap.ncbi.nlm.nih.gov/downloads/frequencies/2010-08_phaseII+III/). We estimated the effective number of tests in admixture mapping using autocorrelation of local ancestry to be 373.1 (Shriner et al., 2011b), yielding a partial Bonferroni-corrected genome-wide significance level $\alpha =\frac{0.05}{373.1}=1.34\times 10^{-4}$. Principal components analysis of the genotype data revealed one significant principal component, which represented two-way admixture (Shriner, 2011). All quantitative phenotypes were Box-Cox-transformed to reduce non-normality and winsorized at ±3 standard deviations to reduce kurtosis.

The Atherosclerosis Risk in Communities Study (ARIC) is a prospective study of atherosclerosis and cardiovascular disease. We obtained approval for data access from dbGaP (Accession phs000280.v2.p1). We retrieved data from the GENEVA sub-study (phs000090.v2.p1), including phenotype data (pht000114) and genotype data (phg000035). ARIC included 2,600 unrelated African Americans from Forsyth County, North Carolina or Jackson, Mississippi. Genotyping was performed using the Affymetrix Genome-Wide Human SNP Array 6.0, with quality control as described previously (Shriner et al., 2009). Local ancestry was inferred for 570,862 autosomal SNPs (Baran et al., 2012). We estimated the effective number of tests in admixture mapping using autocorrelation of local ancestry to be 226.2, yielding a partial Bonferroni-corrected genome-wide significance level $\alpha =\frac{0.05}{226.2}=2.21\times 10^{-4}$. Principal components analysis of the genotype data revealed one significant principal component, which reflected two-way admixture (Figure S1).

### Estimation of the ancestral similarity matrix

We estimated the ancestral similarity matrix **A** for all unrelated individuals in a study using the local ancestry estimates for all autosomal loci. Let *x*_*ij*_ represent the local ancestry, i.e., 0, 1, or 2 chromosomes of African ancestry for the *j*^th^ of *M* individuals at the *i*^th^ of *N* loci. For the *j*^th^ individual, the genome-wide average of local ancestry $\frac{1}{2N}\overset{N}{\underset{i=1}{\sum }}x_{ij}$ is known as global ancestry or the individual admixture proportion. For the HUFS data set, the mean global ancestry was 79.9% ± 11.6%. Similarly, for the ARIC data set, the mean global ancestry was 82.2% ± 10.3%. At the *i*^th^ locus, let $p_{i}=\frac{1}{2M}\overset{M}{\underset{j=1}{\sum }}x_{ij}$. As expected, the trace of ancestry by locus is nearly constant across the autosomes (Figure S2), indicating robustness to natural selection acting at specific loci.

We consider three estimators of pairwise ancestral similarity. First, at a causal locus *i*, we can estimate pairwise ancestral similarity between the *j*^th^ and *k*^th^ individuals based on identity in state:

$$A_{jk}=\{\begin{array}{ccc} \begin{array}{l} \;0.0\text{ if}\\ \;0.5\text{ if}\\ \;1.0\text{ if} \end{array} & \left|\begin{array}{l} x_{ij}-x_{ik}\\ x_{ij}-x_{ik}\\ x_{ij}-x_{ik} \end{array}\right| & \begin{array}{l} =\;2\\ =\;1\\ =\;0. \end{array} \end{array}$$

Second, we can estimate pair-wise ancestral similarity for use in GCTA (Yang et al., 2011a) as

$$A_{jk}=\{\begin{array}{c} \frac{1}{N}\overset{N}{\underset{i=1}{\sum }}\frac{\left(x_{ij}\;-\;2p_{i})(x_{ik}\;-\;2p_{i}\right)}{2p_{i}(1\;-\;p_{i})},j\neq k\\ 1+\frac{1}{N}\overset{N}{\underset{i=1}{\sum }}\frac{x_{ij}^{2}\;-\;(1+2p_{i})x_{ij}\;+\;2p_{i}^{2}}{2p_{i}(1\;-\;p_{i})},j=k. \end{array}$$

For both studies, the number of genotyped SNPs is more than sufficient to yield 100% coverage of switches in local ancestry in African Americans (Shriner et al., 2011a), providing 100% coverage of chromosomal segments and all genetic variation therein. Therefore, corrections for linkage disequilibrium used for genotype data are unnecessary with local ancestry data.

Third, we can estimate pair-wise ancestral similarity as $A_{jk}=\frac{1}{N}\overset{N}{\underset{i=1}{\sum }}\left(x_{ij}-2p_{i}\right)\left(x_{ik}-2p_{i}\right)$. We then estimate the proportion of phenotypic variance explained by local ancestry using GEMMA (Zhou and Stephens, 2012). It is important to note that estimation of similarity in GCTA includes centering by 2*p*_*i*_ and scaling by 2*p*_*i*_(1 − *p*_*i*_); this scaling induces an inverse relationship between *p*_*i*_ and effect size (Speed et al., 2012). In contrast, our estimation of similarity using GEMMA includes centering but not scaling, which *a priori* is more appropriate given that mean local ancestry estimates do not follow an exponential distribution as do allele frequencies but are expected to follow a uniform distribution (Figure S2).

### Simulation to assess bias

To investigate bias in the random effects models implemented in GCTA and GEMMA, we simulated phenotype data based on the white blood cell count data from ARIC. Phenotype data were simulated as the sum of signal normally distributed with mean 0 and variance σ^2^ = 2*p*(1 − *p*)β^2^, with *p* equaling the mean local ancestry at rs2814778 in the ARIC data and β equaling the effect size under the additive model, and random noise normally distributed with mean 0 and variance 1 − σ^2^. We tested for bias using the one-sample Wilcoxon signed rank test.

### Software

GCTA is available at http://www.complextraitgenomics.com/software/gcta/. GEMMA is available at http://www.xzlab.org/software.html.

## Results

**Figure 1:**
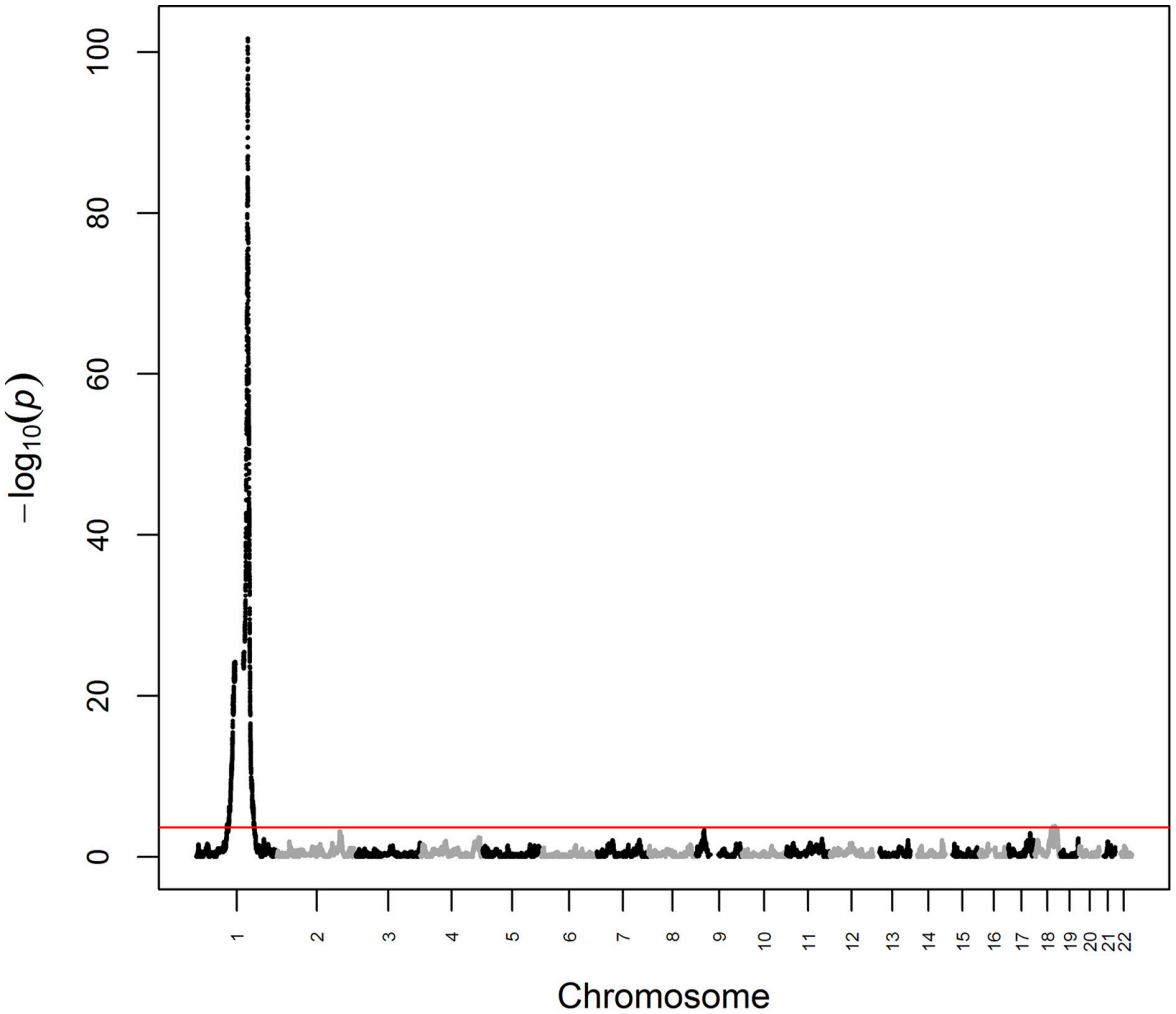
**Manhattan plot from admixture mapping for white blood cell count in ARIC**. White blood cell count was regressed on local ancestry, adjusted for age, global ancestry, sex, and center. The red line indicates the genome-wide significance level.

We first analyzed white blood cell count data from ARIC as a positive control phenotype, i.e., a phenotype for which the genetic architecture is known to include a major ancestry effect. Previous admixture mapping for white blood cell count has revealed a major effect gene at chromosome 1q23 explaining ~20.4% of phenotypic variance (Nalls et al., 2008), with association subsequently mapped to rs2814778 (Reich et al., 2009), a promoter-null variant for the gene *ACKR1* (formerly known as *DARC*) that causes the Fy-/- phenotype. Note that white blood cell count is not unique for having a major effect gene in admixture mapping of African Americans, as rs73885319 in the gene *APOL1* defines a major effect for end-stage kidney disease (Kao et al., 2008; Kopp et al., 2008; Genovese et al., 2010). White blood cell count was Box-Cox-transformed to reduce non-normality and winsorized at ±3 standard deviations to reduce kurtosis. We then performed admixture mapping using linear regression of transformed white blood cell count on local ancestry with age and global ancestry as continuous covariates and sex and study center as discrete covariates. Using this fixed effects model, we estimated that the chromosome 1q23 locus explained 19.3% (*p* = 2.07 × 10^−102^) of the phenotypic variance of white blood cell count (Figure 1). We observed a second genome-wide significant admixture peak (*p* = 1.43 × 10^−4^) on chromosome 18 that explained 0.6% of the phenotypic variance (Figure 1). Taken together, genome-wide significant admixture peaks explained 19.9% of the phenotypic variance of white blood cell count.

To account for the remaining admixture signal that did not reach genome-wide significance, we adapted a random effects model for estimating phenotypic variance explained by common SNPs (Yang et al., 2011a). We performed restricted maximum likelihood analysis of the adjusted white blood cell counts, with age and global ancestry as continuous covariates and sex and study center as discrete covariates. Using GCTA, the proportion of phenotypic variance explained by local ancestry was 16.1%, an underestimate compared to 19.9% obtained by conventional admixture mapping. This result suggests that 19.9% is an overestimate and/or 16.1% is an underestimate.

**Figure 2:**
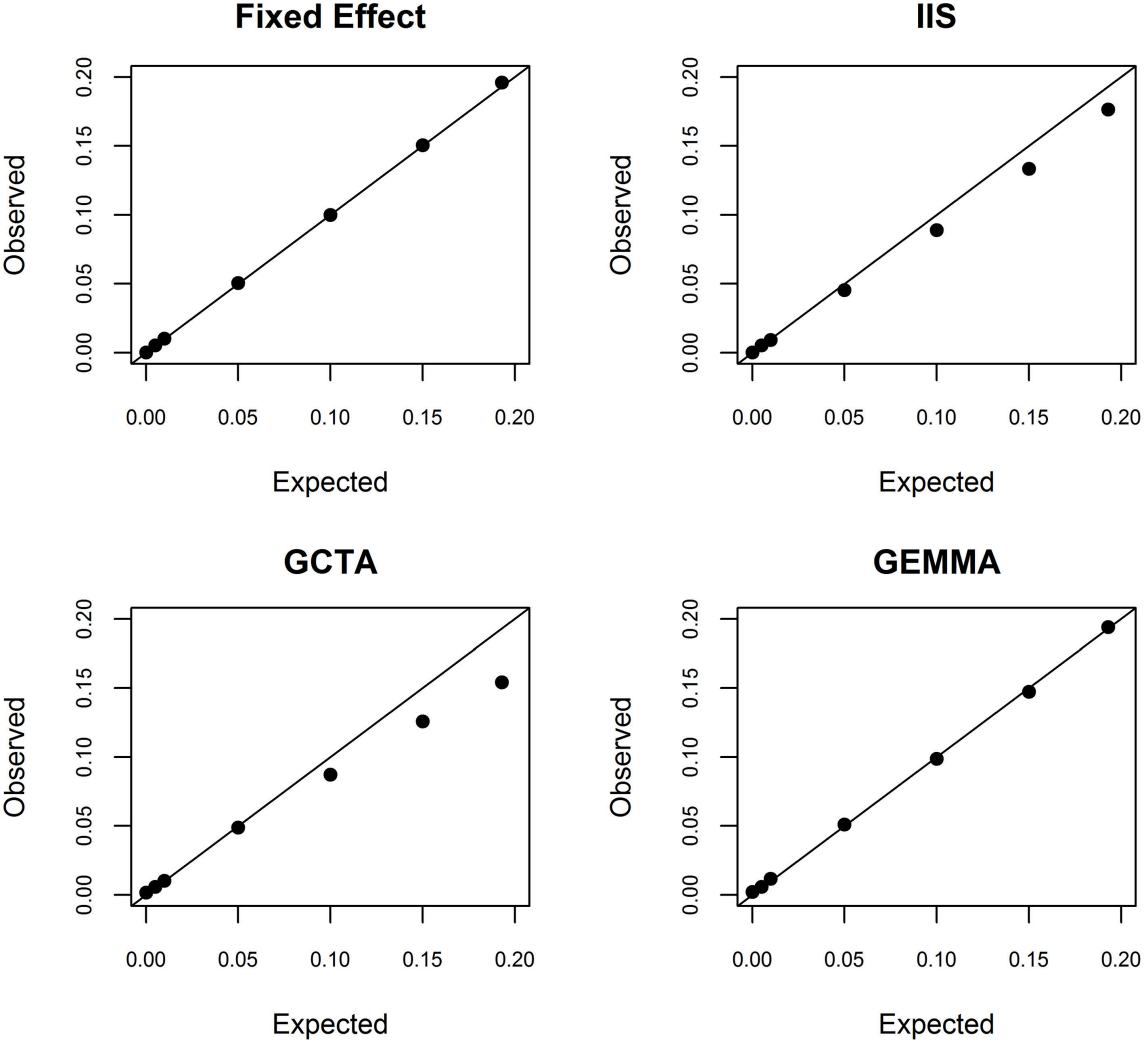
**Simulation study of bias in the random effects model**. Conditional on local ancestry at rs2814778, we simulated a continuous phenotype with a known proportion of phenotypic variance explained by a single causal locus and the remainder of the phenotypic variance being random noise. We randomly generated 100 independent data sets. We then used the fixed effects model (top left), ancestral similarity defined by identity in state (top right), centered and scaled ancestral similarity as defined by GCTA (bottom left), and centered ancestral similarity as defined by GEMMA (bottom right) to estimate the proportion of phenotypic variance explained by local ancestry using similarity estimated genome-wide.

To better understand this estimation problem, we first investigated whether the fixed effects model used in conventional admixture mapping yielded an overestimate. We simulated a quantitative trait conditional on the inferred ancestry states at rs2814778, with a range of additive effect sizes. As theoretically expected, the fixed effects model was unbiased across the entire range of effect sizes (Figure 2). Furthermore, we estimated the conditional power to detect a locus explaining 19.3% of the phenotypic variance to be >99.99%, indicating that the effect size is not overestimated due to the winner's curse. Taken together, we conclude that the estimate of 19.9% phenotypic variance explained is not an overestimate.

We next investigated whether the random effects models suffer from underestimation. It is important to recognize that underestimation of large effect sizes by random effects models compared to fixed effects models is theoretically expected because random effects are assumed to be normally distributed with finite prior variance whereas fixed effects are assumed to be normally distributed with infinite prior variance. Such downward bias has been noted previously for the large effects of HLA on autoimmune diseases (Kang et al., 2010). We investigated this bias in the context of local ancestry analysis by simulating phenotype data given a range of additive effect sizes and conditioned on the local ancestry values at rs2814778. Using simple identity in state, we observed significant underestimation when the proportion of phenotypic variance explained exceeded 1% (Figure 2). Using the centered and scaled estimator of GCTA, we observed significant overestimation when the proportion of phenotypic variance explained was 0% (as expected due to the lower bound of variance at 0), statistically unbiased estimation when the proportion of phenotypic variance explained was between 0 and 5%, and significant underestimation when the proportion of phenotypic variance explained exceeded 10% (Figure 2). Thus, for white blood cell count, the random effects-based estimate from GCTA is systematically biased downward. In contrast, using the centered but not scaled estimator in GEMMA yielded unbiased results, except at the boundary of 0% (Figure 2).

We further estimated similarity using the chromosome and the locus, as would be done in the mapping procedure called genome partitioning (Yang et al., 2011b). Using the centered and scaled estimator, downward bias was exacerbated as similarity was estimated genome-wide down to the causal locus (Table 1), reflecting the fact that unrelated individuals are not unrelated at a shared causal locus. Similarly, using the centered but not scaled estimator, downward bias was also observed but smaller in magnitude (Table 2). Therefore, genome partitioning using either definition of similarity is a biased mapping procedure.

**Table 1:** **Bias in genome partitioning using GCTA**.

**Phenotypic variance explained**	**Genome**		**Chromosome**		**Locus**	
	**Bias**	***P*-value**	**Bias**	***P*-value**	**Bias**	***P*-value**
0.000	0.002	5.77 × 10^−19^	0.001	2.85 × 10^−19^	0.002	2.49 × 10^−19^
0.005	0.001	0.716	0.000	0.938	0.002	0.953
0.010	0.000	0.694	0.000	0.639	0.002	0.712
0.050	−0.001	0.156	−0.004	8.20 × 10^−5^	−0.028	4.20 × 10^−18^
0.100	−0.013	3.07 × 10^−12^	−0.025	1.20 × 10^−17^	−0.073	3.96 × 10^−18^
0.150	−0.024	3.01 × 10^−17^	−0.051	3.96 × 10^−18^	−0.118	3.96 × 10^−18^
0.193	−0.039	4.08 × 10^−18^	−0.079	3.96 × 10^−18^	−0.158	3.96 × 10^−18^

**Table 2:** **Bias in genome partitioning using GEMMA**.

**Phenotypic variance explained**	**Genome**		**Chromosome**		**Locus**	
	**Bias**	***P*-value**	**Bias**	***P*-value**	**Bias**	***P*-value**
0.000	0.002	3.24 × 10^−19^	0.001	5.19 × 10^−19^	0.002	3.89 × 10^−20^
0.005	0.001	0.706	0.001	0.551	0.008	2.68 × 10^−9^
0.010	0.002	0.172	0.001	0.756	0.024	2.27 × 10^−10^
0.050	0.001	0.985	0.002	0.391	0.028	1.05 × 10^−7^
0.100	−0.001	0.224	0.006	7.82 × 10^−3^	−0.006	6.11 × 10^−3^
0.150	−0.003	0.136	0.008	1.43 × 10^−3^	−0.045	7.67 × 10^−17^
0.193	0.001	0.493	0.011	2.92 × 10^−4^	−0.071	2.11 × 10^−17^

We then surveyed genome-wide variance explained by local ancestry using both GCTA and GEMMA for 25 phenotypes with data in both the ARIC and HUFS data sets: height, weight, body mass index, waist circumference, hip circumference, and waist-hip ratio; type 2 diabetes, fasting insulin, and fasting glucose; hypertension, systolic blood pressure, and diastolic blood pressure; total cholesterol, high density lipoprotein, low density lipoprotein, and triglycerides; creatinine and the estimated glomerular filtration rate; and albumin, calcium, C-reactive protein, potassium, sodium, total protein, and uric acid. Using the ARIC data set, we first performed analysis of the transformed phenotypic data, with age and global ancestry as continuous covariates and sex and study center as discrete covariates. The estimates of the proportions of phenotypic variance explained by local ancestry ranged from 0 to 3.8% (Table 3). Using the HUFS data set, we performed analysis with age and global ancestry as continuous covariates and sex as a discrete covariate. The estimates of the proportions of phenotypic variance explained by local ancestry ranged from 0 to 8.1% (Table 3). As expected given the smaller sample size, the standard errors were approximately twice as big for HUFS as for ARIC (Table 3). Whereas one phenotype (sodium) yielded a zero estimate of phenotypic variance explained by local ancestry in ARIC, six phenotypes (C-reactive protein, creatinine, diastolic blood pressure, potassium, sodium, and triglycerides) yielded a zero estimate of phenotypic variance explained by local ancestry in HUFS (Table 3). To confirm these zero estimates, we performed admixture mapping using linear regression. We detected genome-wide significant admixture peaks for sodium (in both ARIC and HUFS) and potassium (Figure S3). Thus, we recommend performing conventional admixture mapping based on fixed effects in conjunction with variance components estimation based on random effects to gain a more complete understanding of genetic architecture.

**Table 3:** **Genome-wide proportion of phenotypic variance explained by local ancestry**.

**Phenotype**	**ARIC (GCTA)**		**ARIC (GEMMA)**		**HUFS (GCTA)**		**HUFS (GEMMA)**	
	**Variance**	**SE**	**Variance**	**SE**	**Variance**	**SE**	**Variance**	**SE**
Height	0.0306	0.0128	0.0265	0.0119	0.0540	0.0303	0.0532	0.0326
Weight	0.0165	0.0113	0.0143	0.0110	0.0157	0.0249	0.0074	0.0238
Body mass index	0.0283	0.0127	0.0256	0.0123	0.0220	0.0256	0.0148	0.0247
Waist circumference	0.0114	0.0106	0.0116	0.0112	0.0370	0.0281	0.0249	0.0262
Hip circumference	0.0263	0.0125	0.0221	0.0118	0.0080	0.0231	0.0046	0.0229
Waist-hip ratio	0.0037	0.0097	0.0023	0.0103	0.0593	0.0313	0.0672	0.0362
Systolic blood pressure	0.0035	0.0089	0.0041	0.0092	0.0038	0.0229	0.0032	0.0234
Diastolic blood pressure	0.0081	0.0098	0.0082	0.0100	0.0000	0.0233	0.0000	0.0191
Hypertension (observed scale)	0.0203	0.0119	0.0207	0.0126	0.0084	0.0230	0.0127	0.0250
Hypertension (liability scale)	0.0322	0.0188	NA	NA	0.0131	0.0359	NA	NA
Fasting glucose	0.0283	0.0131	0.0212	0.0117	0.0293	0.0279	0.0250	0.0290
Fasting insulin	0.0049	0.0092	0.0039	0.0087	0.0057	0.0231	0.0096	0.0247
Type 2 diabetes (observed scale)	0.0173	0.0115	0.0146	0.0112	0.0324	0.0277	0.0273	0.0280
Type 2 diabetes (liability scale)	0.0247	0.0164	NA	NA	0.0809	0.0692	NA	NA
Triglycerides	0.0140	0.0112	0.0117	0.0111	0.0000	0.0254	0.0000	0.0411
High density lipoprotein	0.0292	0.0130	0.0295	0.0133	0.0115	0.0226	0.0171	0.0236
Low density lipoprotein	0.0380	0.0149	0.0328	0.0150	0.0073	0.0230	0.0073	0.0233
Total cholesterol	0.0249	0.0128	0.0236	0.0133	0.0347	0.0265	0.0400	0.0286
Sodium	0.0000	0.0097	0.0000	0.0105	0.0000	0.0215	0.0000	0.0219
Potassium	0.0197	0.0120	0.0156	0.0113	0.0000	0.0234	0.0000	0.0246
Calcium	0.0096	0.0098	0.0069	0.0087	0.0000	0.0231	0.0034	0.0253
Uric Acid	0.0059	0.0095	0.0056	0.0095	0.0000	0.0211	0.0001	0.0203
C-reactive protein	0.0091	0.0130	0.0096	0.0126	0.0000	0.0318	0.0000	0.0387
Albumin	0.0063	0.0098	0.0062	0.0102	0.0028	0.0223	0.0050	0.0231
Total protein	0.0180	0.0112	0.0154	0.0106	0.0604	0.0367	0.0698	0.0405
Creatinine	0.0161	0.0105	0.0159	0.0100	0.0000	0.0218	0.0000	0.0224
Estimated glomerular filtration rate	0.0181	0.0109	0.0173	0.0103	0.0012	0.0217	0.0044	0.0229

For hypertension and type 2 diabetes, we report phenotypic variance explained on the observed binary scale and on the unobserved liability scale, assuming a prevalence of hypertension of 0.44 (Centers for Disease Control and Prevention, 2014) and a prevalence of type 2 diabetes of 0.187 (Centers for Disease Control and Prevention, 2011).

## Discussion

We surveyed 26 quantitative traits and disease outcomes, mostly anthropometric and metabolic, to understand the proportion of phenotypic variance explained by local ancestry in admixed African Americans. We used an extension of linear mixed models in which genetic similarity was defined in terms of local ancestry rather than genotype. In two large independent samples of unrelated African Americans, we found that local ancestry at major and polygenic effect genes can explain up to 20 and 8% of phenotypic variance, respectively.

Theoretically, for a purely polygenic trait in an admixed population, the proportion of additive genetic variance explained by local ancestry is determined by the mixture proportions and the amount of genetic differentiation among the parental populations (Zaitlen et al., 2014). Given a two-way admixed population with a mixture proportion θ and genetic differentiation between the parental populations *F*_*ST*_, a fraction 2*F*_*ST*_θ(1 − θ) of the additive genetic variance is variance due to local ancestry (Zaitlen et al., 2014). To illustrate, in an admixed African American population with 80% African and 20% European ancestry, assuming *F*_*ST*_ = 0.058 (The 1000 Genomes Project Consortium, 2012), this fraction is 1.9%. For traits with genetic architectures containing large effect genes, this fraction could be substantially higher, as we demonstrated for white blood cell count. However, Zaitlen et al. (2014) implicitly assume that genotype effect sizes are constant across ancestries and that only differences in allele frequencies contribute to ancestry effects. Consequently, their estimation of additive genetic variance requires estimates of genotype effect sizes with confounding by local (and global) ancestry removed. Also, by ignoring loci at which genotype effects differ by ancestry, their estimation of additive genetic variance potentially misestimates heritability. As a practical note, we have shown that centered but unscaled ancestral similarity is more appropriate than centered and scaled ancestral similarity, so that their estimates of variance explained by local ancestry based on GCTA are likely underestimates.

There are limitations of the random effects approach to estimating the polygenic variance component. First, we showed that effect size estimation at large effect genes is systematically biased downward. One approach to address this limitation is (1) perform conventional admixture mapping in order to identify loci with large effects and (2) model these loci using fixed effects rather than random effects in a mixed effects model (Kang et al., 2010; Segura et al., 2012). Another approach is to jointly estimate separate terms for the polygenic component and the additional effects of loci with larger effects all in one model (Rakitsch et al., 2013; Zhou et al., 2013; Loh et al., 2015). In either approach, ancestral similarity should not be standardized by the empirical variance. Second, we showed that genome partitioning is biased. This bias occurs because unrelated individuals are not unrelated at a shared causal locus. Third, sampling error is ignored in the estimation of the similarity matrix (Yang et al., 2010). Errors in local ancestry inference could adversely affect estimation of proportions of phenotypic variance explained by local ancestry. This type of error has not been found to be problematic for African Americans, for which local ancestry inference is highly accurate, but could be a problem for other admixed populations (Zaitlen et al., 2014). Fourth, similarity is currently only defined for two-way admixture. The extension of estimation of the proportion of phenotypic variance explained by local ancestry in the situation of multi-way admixture is straightforward in principle. The only procedural difference is to use an estimator of ancestral similarity that accounts for more than two ancestries. By analogy with multi-allelic markers such as microsatellites, there are several distance measures that could be considered. For example, Smouse and Peakall (1999) suggested that homozygotes in a diploid system of three codominant alleles could be represented by the vertices of an equilateral triangle, with the heterozygotes positioned midway between the respective homozygotes (Table 4). Kosman and Leonard (2005) criticized this geometric model on the grounds that there is no genetic reason why homozygotes *AA* and *BB* should be more distant than homozygote *AA* and heterozygote *BC*. Instead, they suggested defining 100% identity in state if both alleles are shared, 50% identity in state if one allele is shared, and 0% identity in state if no alleles are shared (Table 4). Another consideration is whether distances should be weighted, perhaps by the genetic distance between ancestries (Morris, 2011).

**Table 4:** **Genetic distance assuming diploidy and three codominant alleles**.^*^

	***AA***	***AB***	***AC***	***BB***	***BC***	***CC***
*AA*	0	1	1	2	2	2
*AB*	1	0	1	1	1	2
*AC*	1	1	0	2	1	1
*BB*	2	1	$\sqrt{3}$	0	1	2
*BC*	$\sqrt{3}$	1	1	1	0	1
*CC*	2	$\sqrt{3}$	1	2	1	0

* The Euclidean distance-based model of Smouse and Peakall (1999) is below the diagonal. The Hamming distance-based model of Kosman and Leonard (2005) is above the diagonal. In both models, AB, AC, and BC are assumed to be identical to BA, CA, and CB, respectively.

Our results can be interpreted from several perspectives. One, the proportion of phenotypic variance explained by local ancestry is a direct measure of the proportion of phenotypic variance amenable to admixture mapping. A major implication of our results is that admixture mapping can benefit from a linear mixed effects model including the polygenic effect of local ancestry. Two, it is generally unknown how much health disparities reflect genetic vs. socio-economic or environmental factors. Our results inform this issue by providing estimates of the fraction of genetic factors that contribute to ancestry-level differences for multiple phenotypes. For example, given that the expected fraction of cases in a population-based study is equal to prevalence, our estimates of the phenotypic variance explained by local ancestry for hypertension and type 2 diabetes directly assess ancestry effects on prevalence, thereby directly addressing a major measure of health disparities. How much these ancestry-level differences ultimately contribute to health disparities remains unknown because the corresponding proportion of all non-genetic sources of phenotypic variance that affect differences in prevalence or other health disparities is unknown. Three, our results illuminate the magnitude of error resulting from association studies not controlling for local ancestry, while also revealing the extent to which phenotype-associated SNPs have cosmopolitan vs. population-specific effects.

## Funding

The Atherosclerosis Risk in Communities (ARIC) Study was carried out as a collaborative study supported by National Heart, Lung, and Blood Institute contracts HHSN268201100005C, HHSN268201100006C, HHSN268201100007C, HHSN268201100008C, HHSN268201100009C, HHSN268201100010C, HHSN268201100011C, and HHSN268201100012C. Funding for the ARIC Gene Environment Association Studies (dbGaP Study Accession phs000090.v2.p1, a sub-study of phs000280.v2.p1) was provided by National Human Genome Research Institute grant U01HG004402 (Eric Boerwinkle). Support for the Howard University Family Study was provided by National Institutes of Health grants S06GM008016-320107, S06GM008016-380111, and 2M01RR010284. Genotyping support was provided by the Coriell Institute for Medical Research. This research was supported by the Intramural Research Program of the Center for Research on Genomics and Global Health (CRGGH). The CRGGH is supported by the National Human Genome Research Institute, the National Institute of Diabetes and Digestive and Kidney Diseases, the Center for Information Technology, and the Office of the Director at the National Institutes of Health (Z01HG200362).

### Conflict of interest statement

The authors declare that the research was conducted in the absence of any commercial or financial relationships that could be construed as a potential conflict of interest.
