# Supplementary material for: Phenotypic variance explained by local ancestry in admixed African Americans
Source: Front Genet. 2015 Oct 29;6:324. doi: 10.3389/fgene.2015.00324 (PMC4625172; doi:10.3389/fgene.2015.00324)
Supplement: Supplementary file 1 [file DataSheet1.PDF]

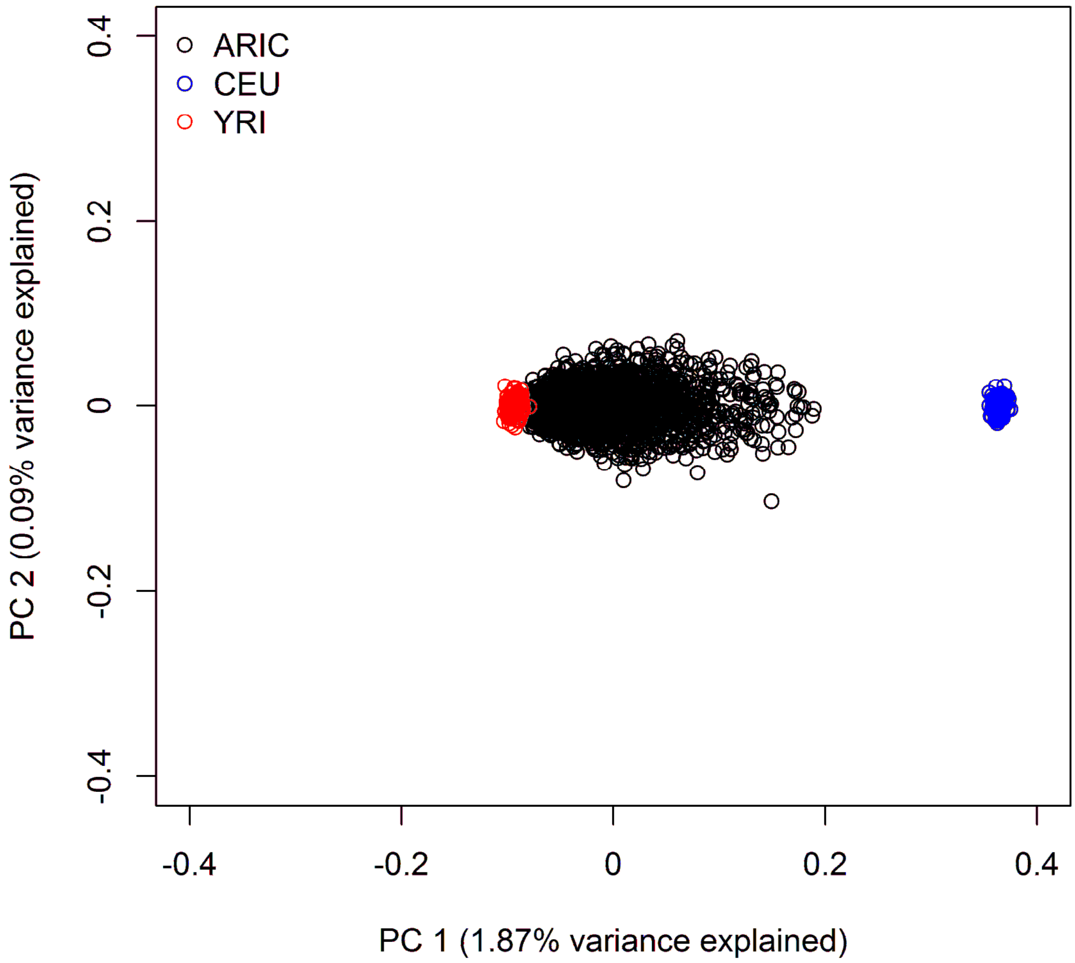

Figure S1. Principal components analysis of the ARIC genotype data. Eigendecomposition of the centered sample covariance matrix was performed on ARIC ($n = 2,600$) merged with the HapMap Phase III CEU ($n = 112$) and YRI ($n = 113$) samples. Using the ARIC genotype data, we removed strand-ambiguous SNPs and then pruned for approximate linkage equilibrium at an r^2 threshold of 0.1, leaving 76,409 SNPs.

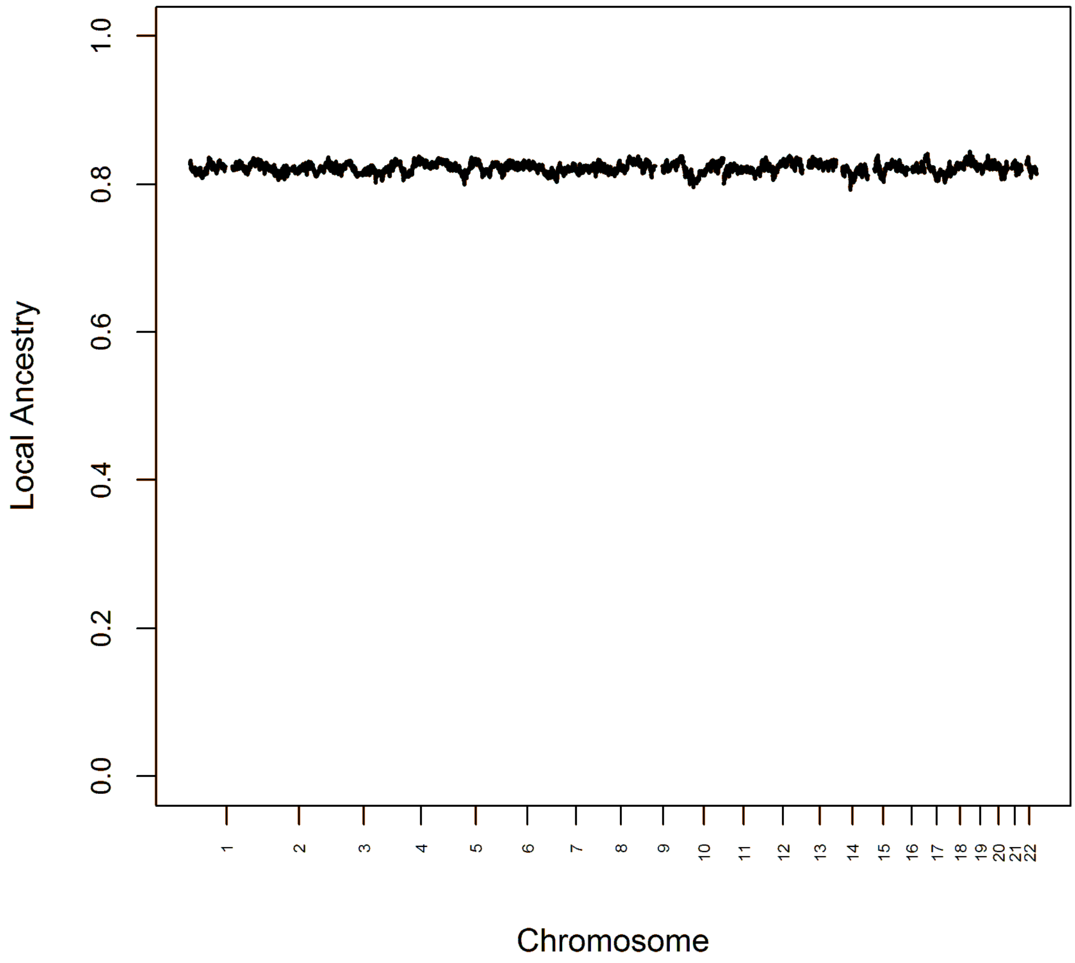

Figure S2. Trace of the mean local ancestry in ARIC.

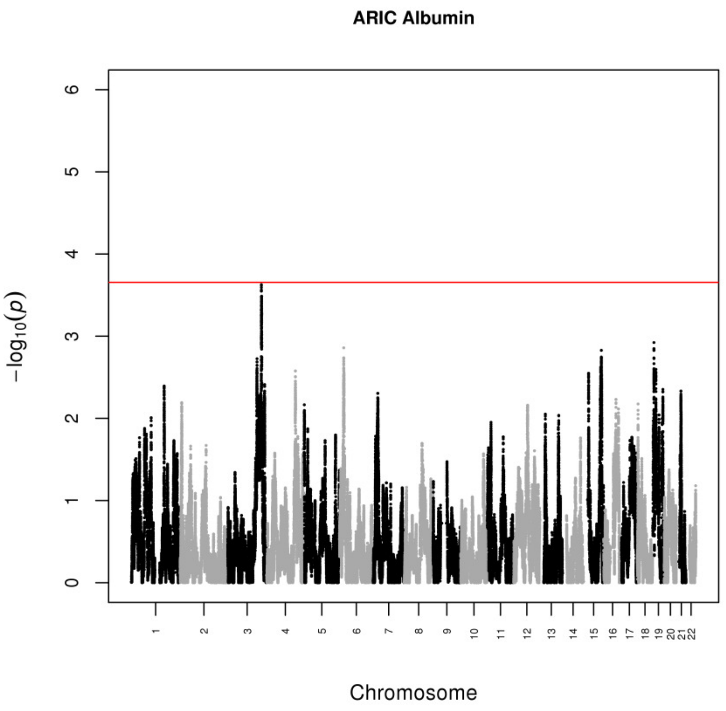
ARIC Albumin
$-\log_{10}(p)$
0
1
2
3
4
5
6
1
2
3
4
5
6
7
8
9
10
11
12
13
14
15
16
17
18
19
20
21
22
Chromosome

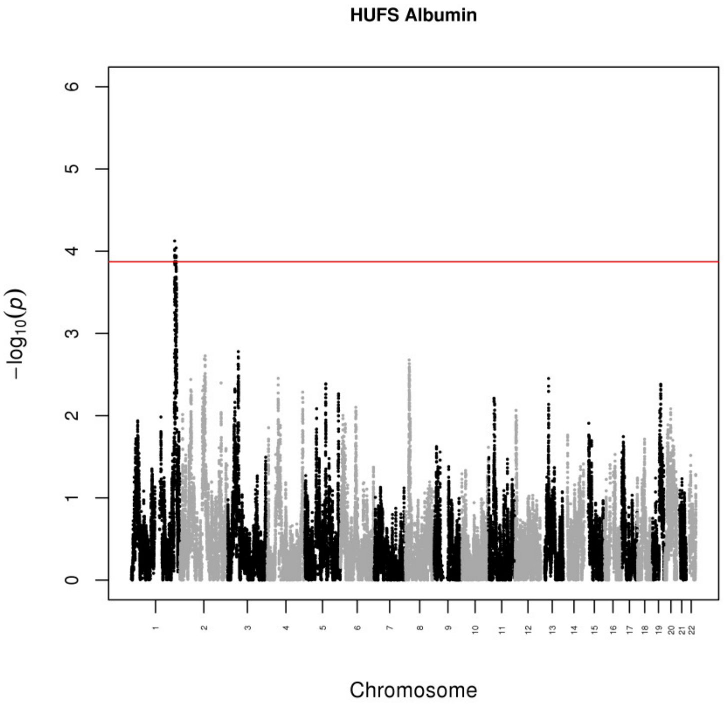
HUFS Albumin
$-\log_{10}(p)$
0
1
2
3
4
5
6
1
2
3
4
5
6
7
8
9
10
11
12
13
14
15
16
17
18
19
20
21
22
Chromosome

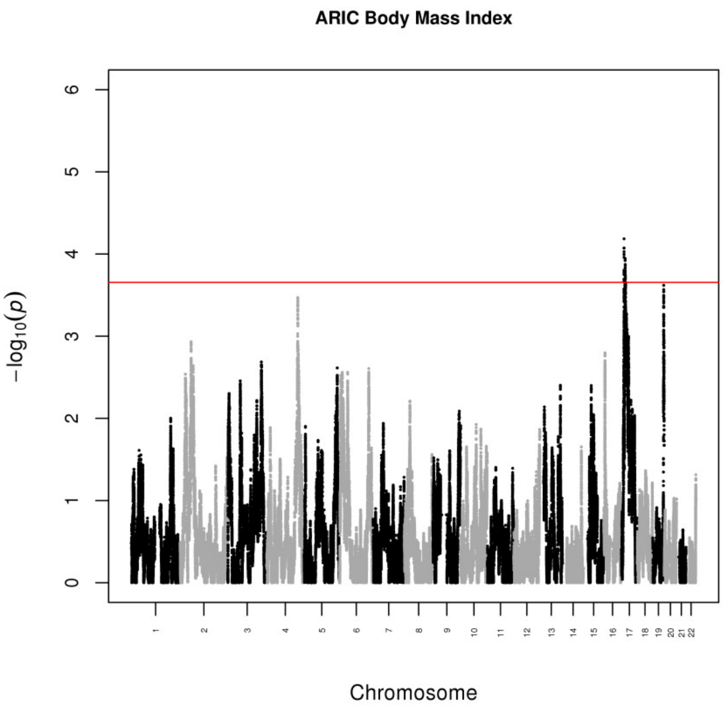
ARIC Body Mass Index
$-\log_{10}(p)$
0
1
2
3
4
5
6
1
2
3
4
5
6
7
8
9
10
11
12
13
14
15
16
17
18
19
20
21
22
Chromosome

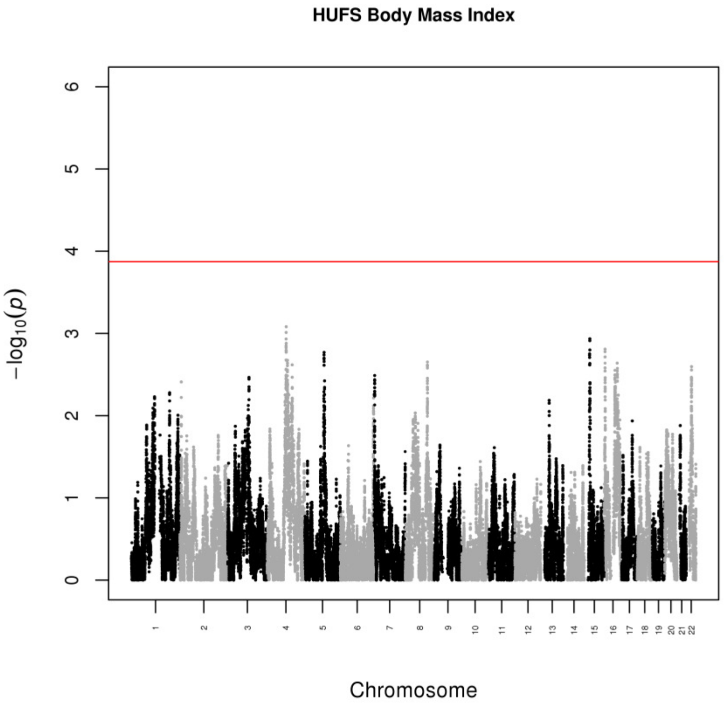
HUFS Body Mass Index
$-\log_{10}(p)$
0
1
2
3
4
5
6
1
2
3
4
5
6
7
8
9
10
11
12
13
14
15
16
17
18
19
20
21
22
Chromosome

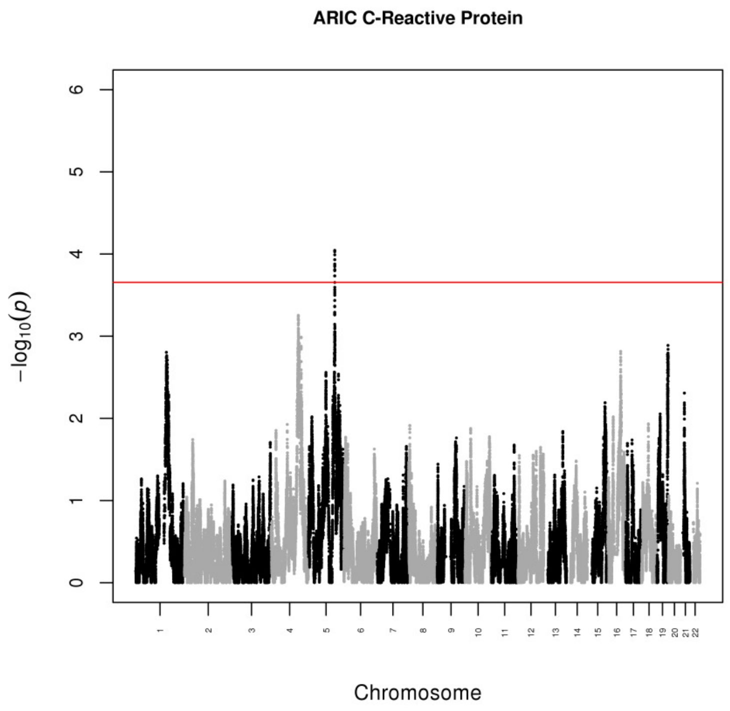
ARIC C-Reactive Protein
6
5
4
3
2
1
0
$-\log_{10}(p)$
1
2
3
4
5
6
7
8
9
10
11
12
13
14
15
16
17
18
19
20
21
22
Chromosome

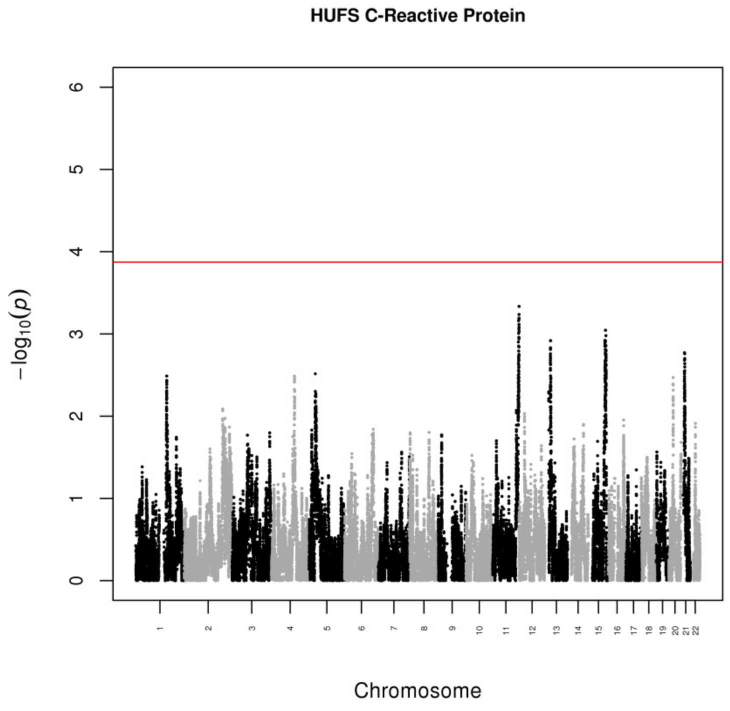
HUFS C-Reactive Protein
6
5
4
3
2
1
0
$-\log_{10}(p)$
1
2
3
4
5
6
7
8
9
10
11
12
13
14
15
16
17
18
19
20
21
22
Chromosome

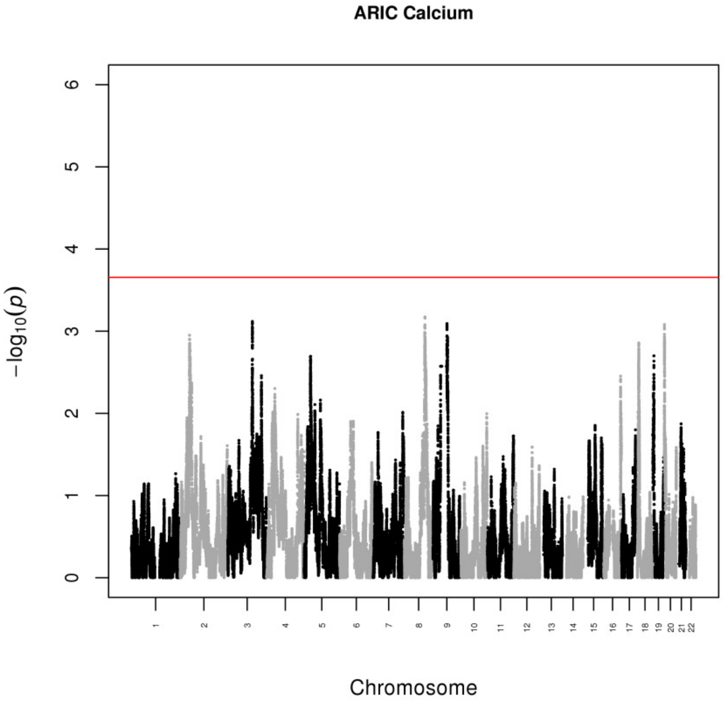
ARIC Calcium
$-\log_{10}(p)$
Chromosome

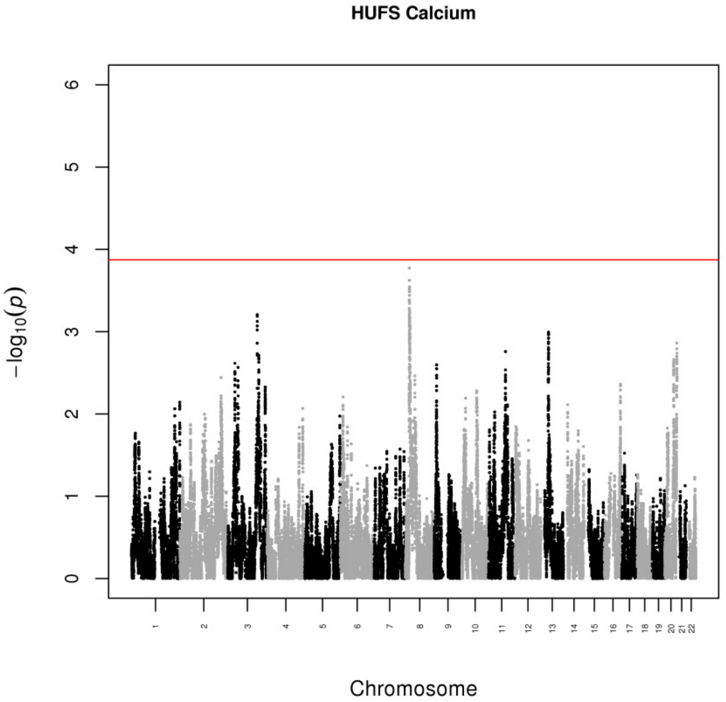
HUFS Calcium
$-\log_{10}(p)$
Chromosome

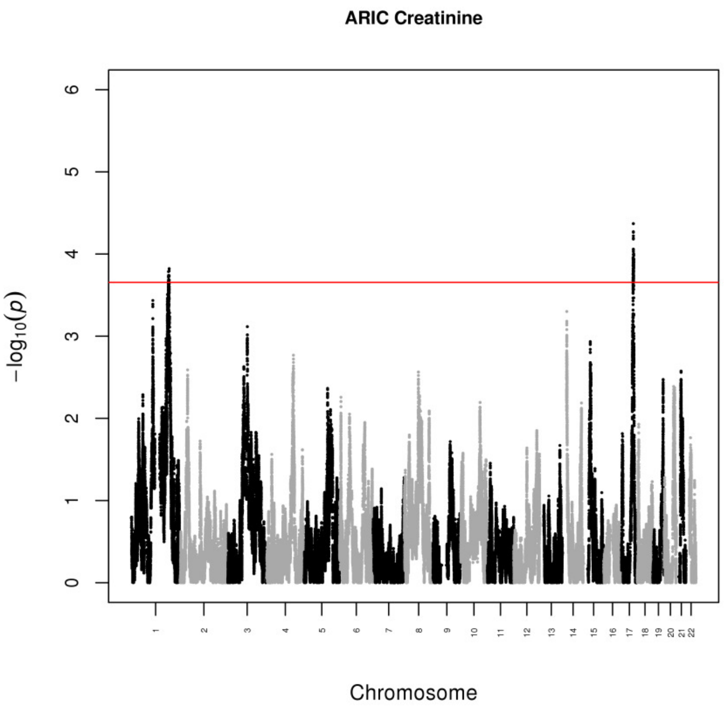
ARIC Creatinine
$-\log_{10}(p)$
0
1
2
3
4
5
6
1
2
3
4
5
6
7
8
9
10
11
12
13
14
15
16
17
18
19
20
21
22
Chromosome

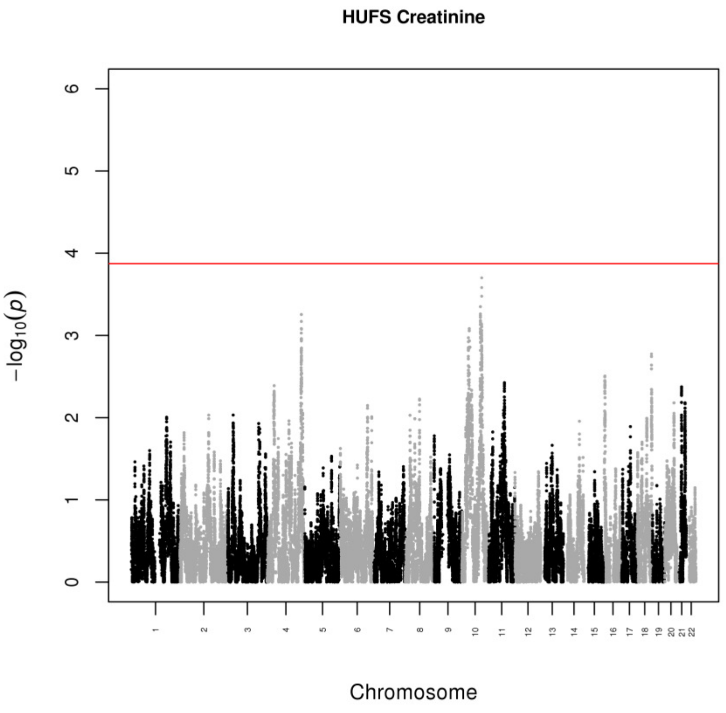
HUFS Creatinine
$-\log_{10}(p)$
0
1
2
3
4
5
6
1
2
3
4
5
6
7
8
9
10
11
12
13
14
15
16
17
18
19
20
21
22
Chromosome

ARIC Diastolic Blood Pressure

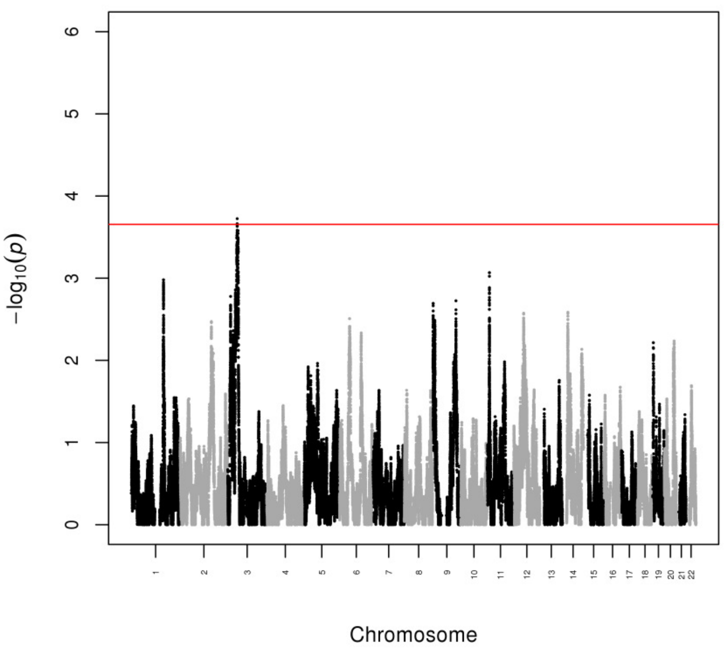

HUFS Diastolic Blood Pressure

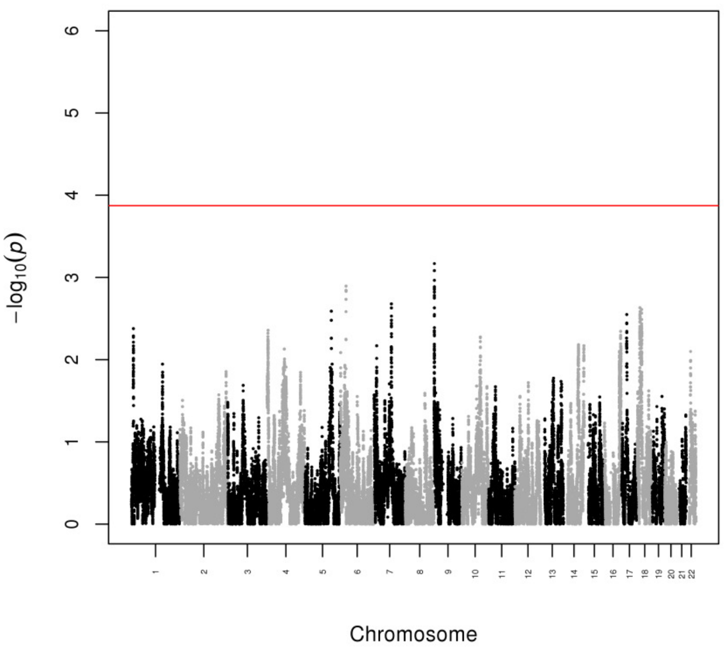

ARIC Estimated Glomerular Filtration Rate

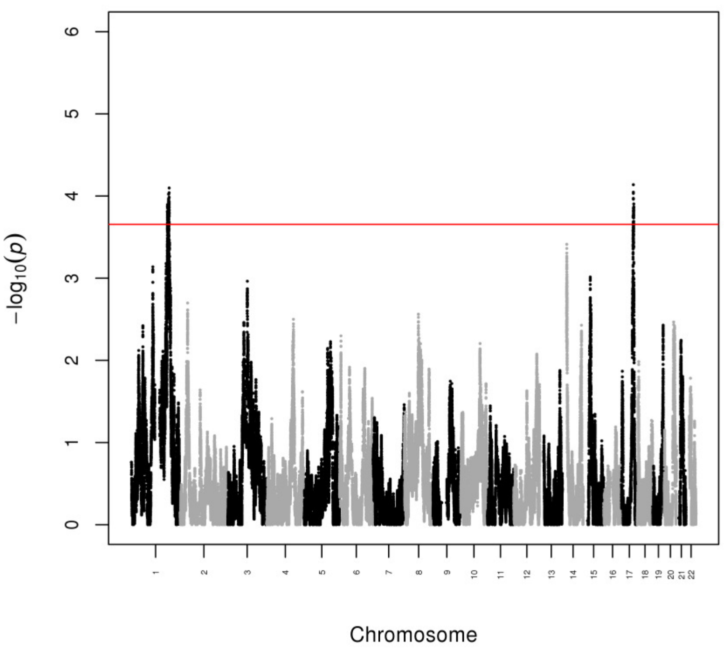

HUFS Estimated Glomerular Filtration Rate

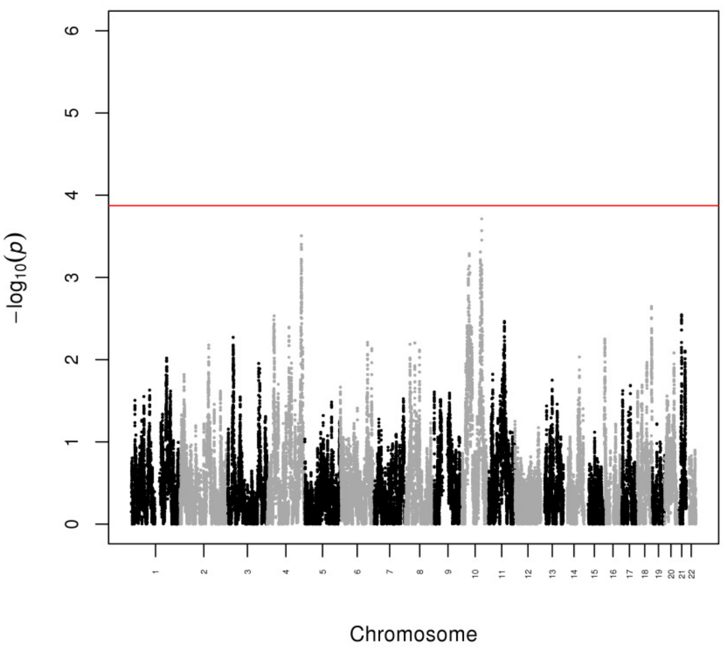

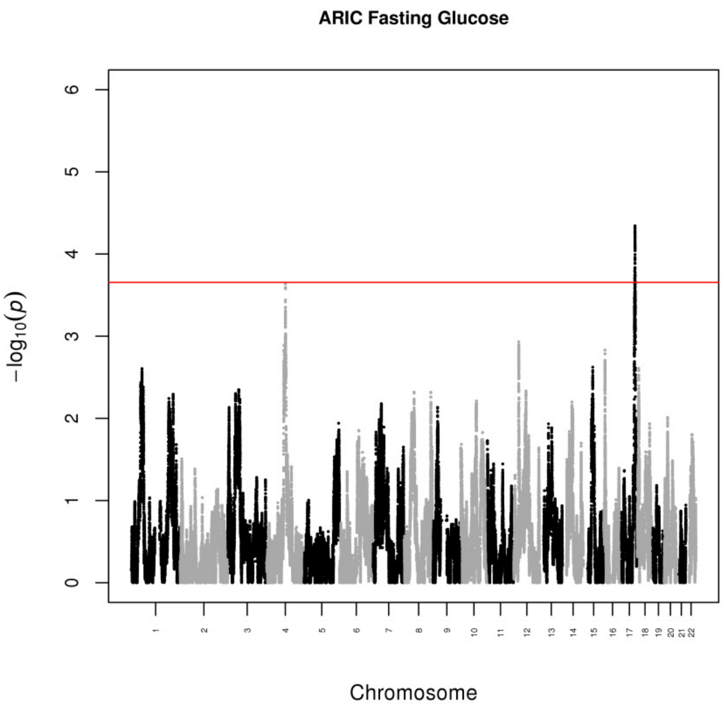
ARIC Fasting Glucose
$-\log_{10}(p)$
0
1
2
3
4
5
6
Chromosome
1
2
3
4
5
6
7
8
9
10
11
12
13
14
15
16
17
18
19
20
21
22

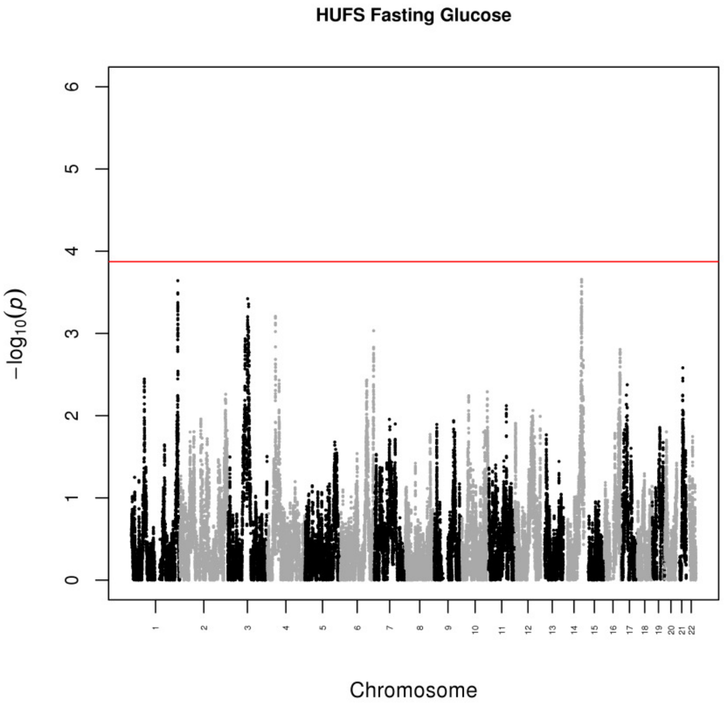
HUFS Fasting Glucose
$-\log_{10}(p)$
0
1
2
3
4
5
6
Chromosome
1
2
3
4
5
6
7
8
9
10
11
12
13
14
15
16
17
18
19
20
21
22

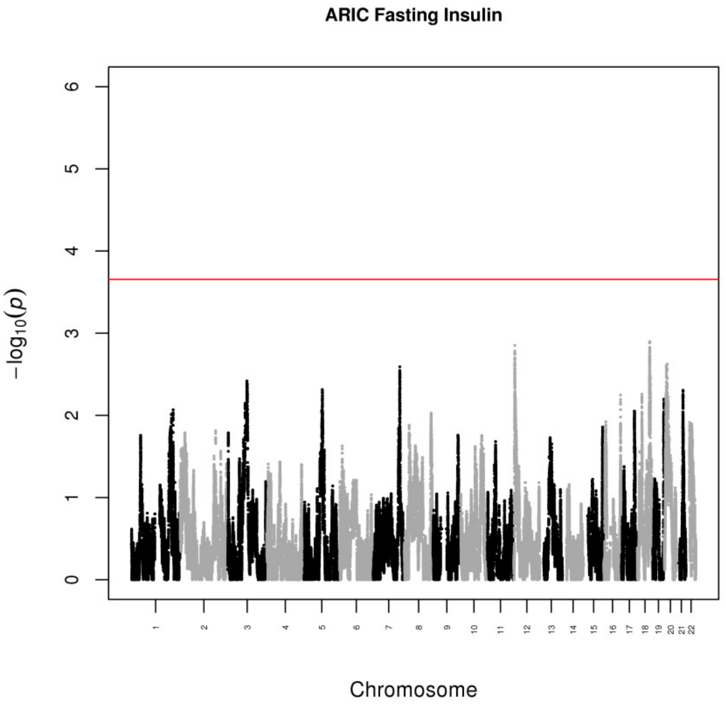
ARIC Fasting Insulin
$-\log_{10}(p)$
0
1
2
3
4
5
6
1 2 3 4 5 6 7 8 9 10 11 12 13 14 15 16 17 18 19 20 21 22
Chromosome

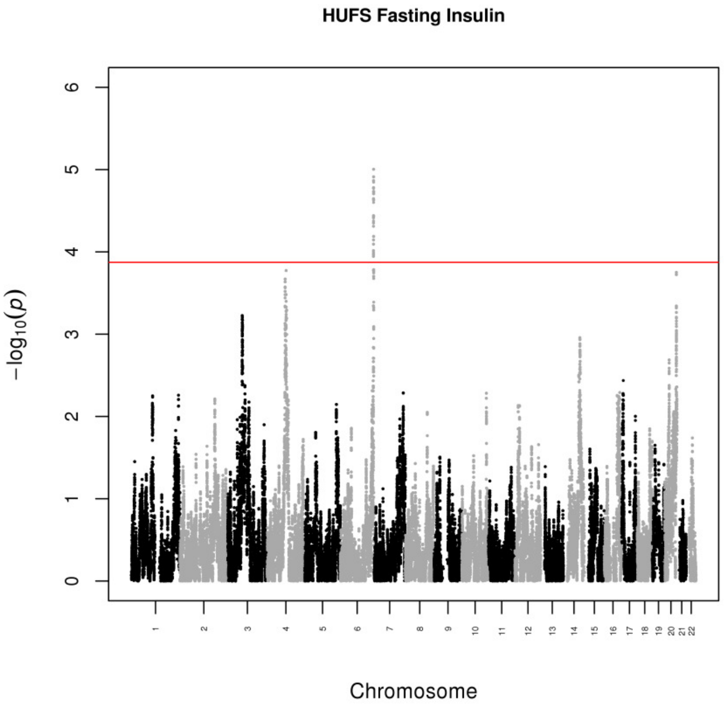
HUFS Fasting Insulin
$-\log_{10}(p)$
0
1
2
3
4
5
6
1 2 3 4 5 6 7 8 9 10 11 12 13 14 15 16 17 18 19 20 21 22
Chromosome

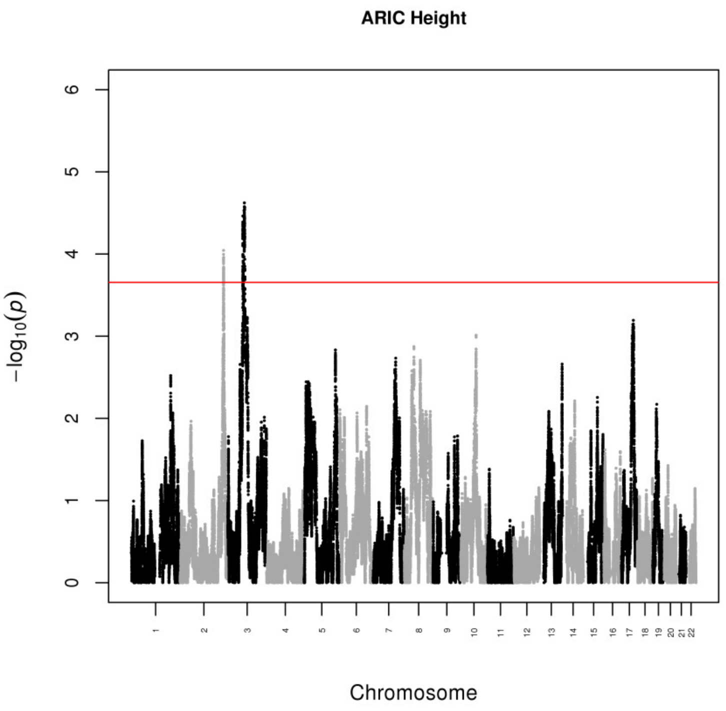
ARIC Height
0
1
2
3
4
5
6
−log10(p)
1
2
3
4
5
6
7
8
9
10
11
12
13
14
15
16
17
18
19
20
21
22
Chromosome

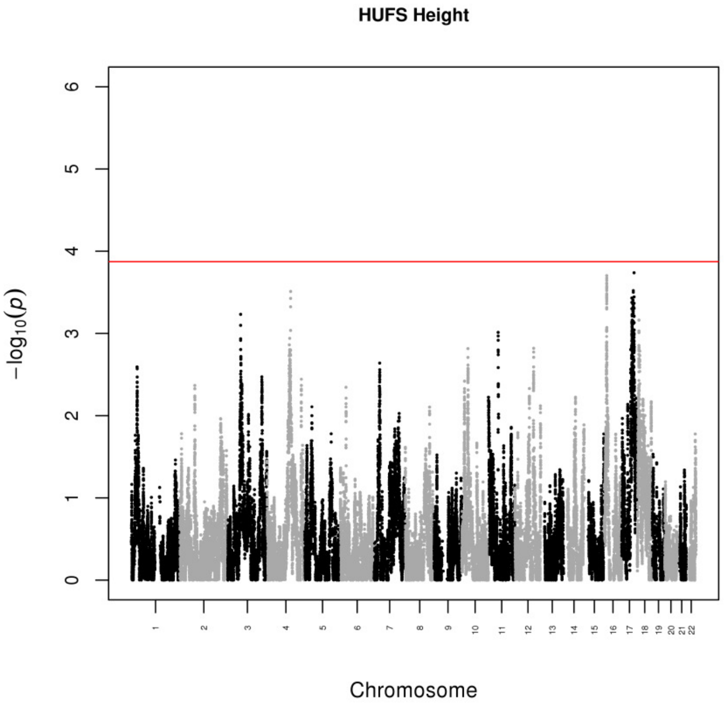
HUFS Height
0
1
2
3
4
5
6
−log10(p)
1
2
3
4
5
6
7
8
9
10
11
12
13
14
15
16
17
18
19
20
21
22
Chromosome

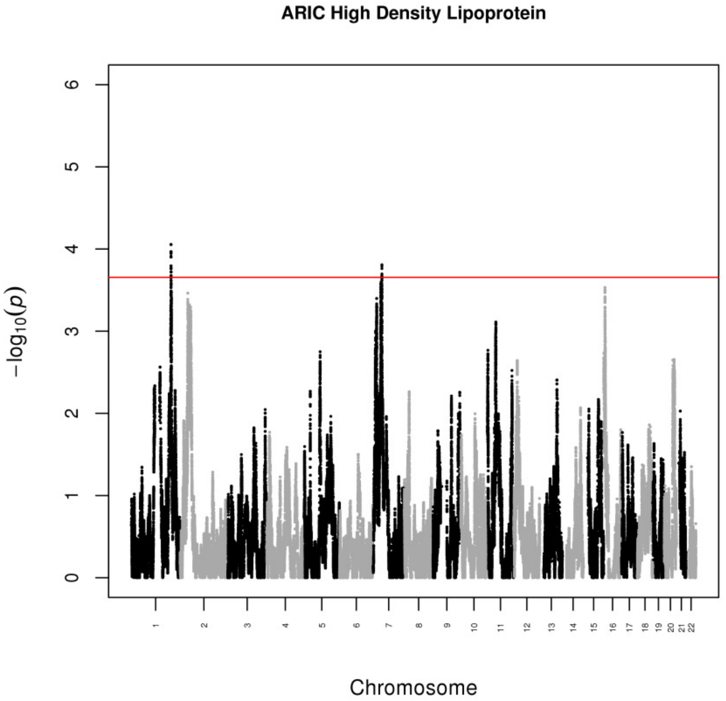
ARIC High Density Lipoprotein
$-\log_{10}(p)$
0
1
2
3
4
5
6
1
2
3
4
5
6
7
8
9
10
11
12
13
14
15
16
17
18
19
20
21
22
Chromosome

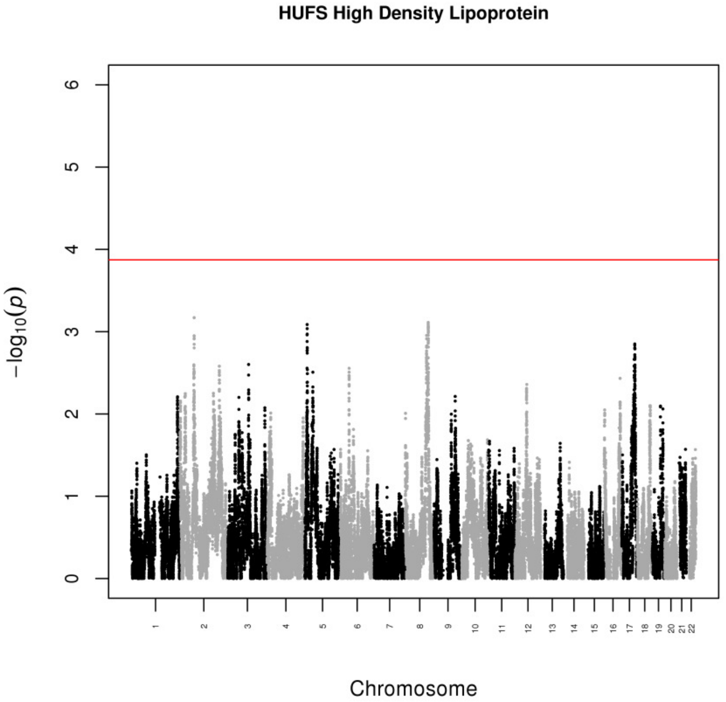
HUFS High Density Lipoprotein
$-\log_{10}(p)$
0
1
2
3
4
5
6
1
2
3
4
5
6
7
8
9
10
11
12
13
14
15
16
17
18
19
20
21
22
Chromosome

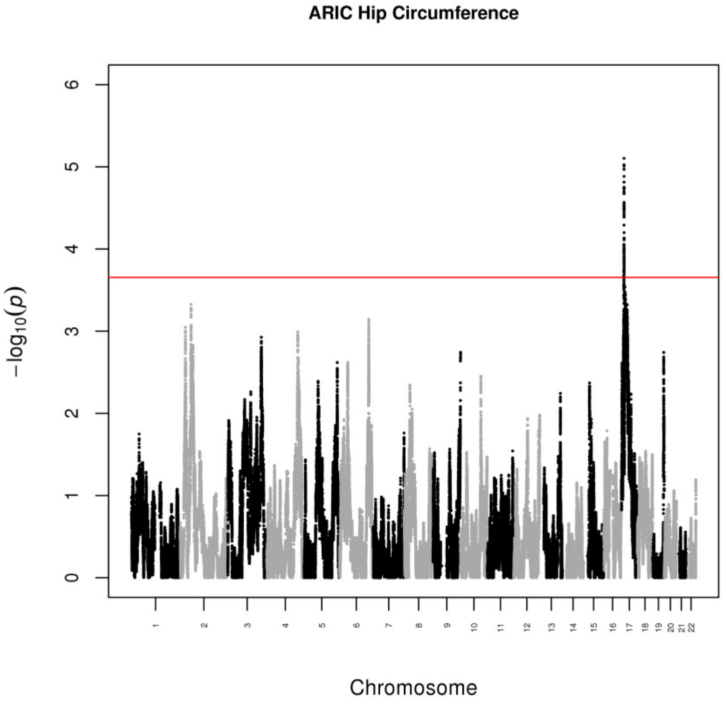
ARIC Hip Circumference
$-\log_{10}(p)$
0
1
2
3
4
5
6
1
2
3
4
5
6
7
8
9
10
11
12
13
14
15
16
17
18
19
20
21
22
Chromosome

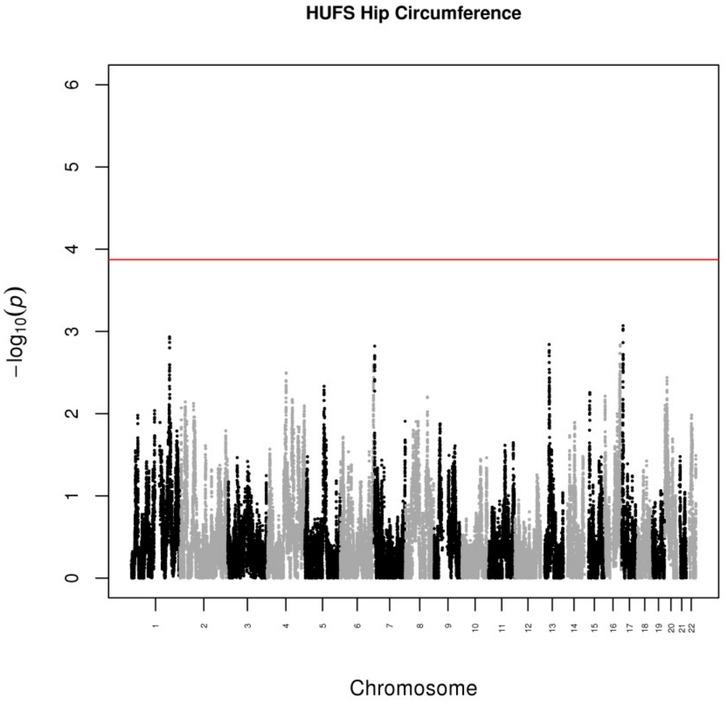
HUFS Hip Circumference
$-\log_{10}(p)$
0
1
2
3
4
5
6
1
2
3
4
5
6
7
8
9
10
11
12
13
14
15
16
17
18
19
20
21
22
Chromosome

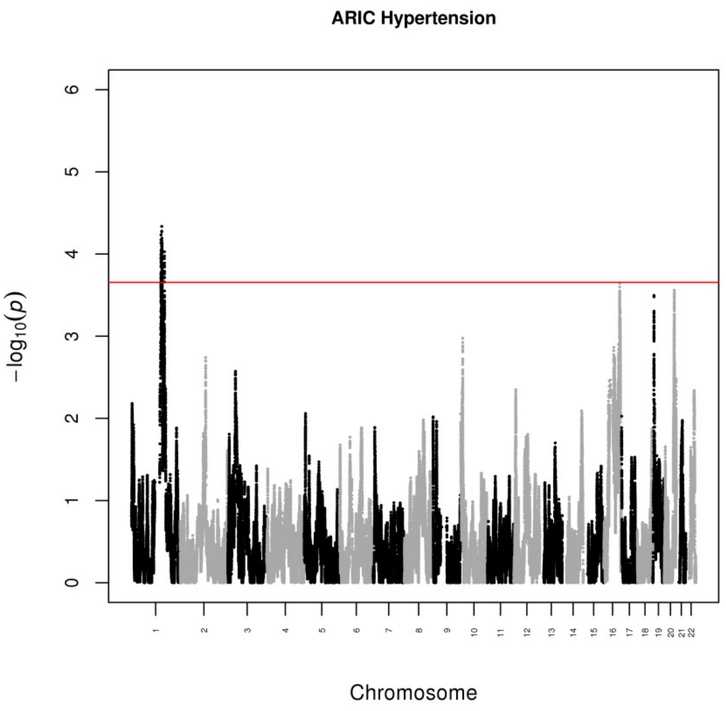
ARIC Hypertension
−log10(p)
0
1
2
3
4
5
6
Chromosome
1
2
3
4
5
6
7
8
9
10
11
12
13
14
15
16
17
18
19
20
21
22

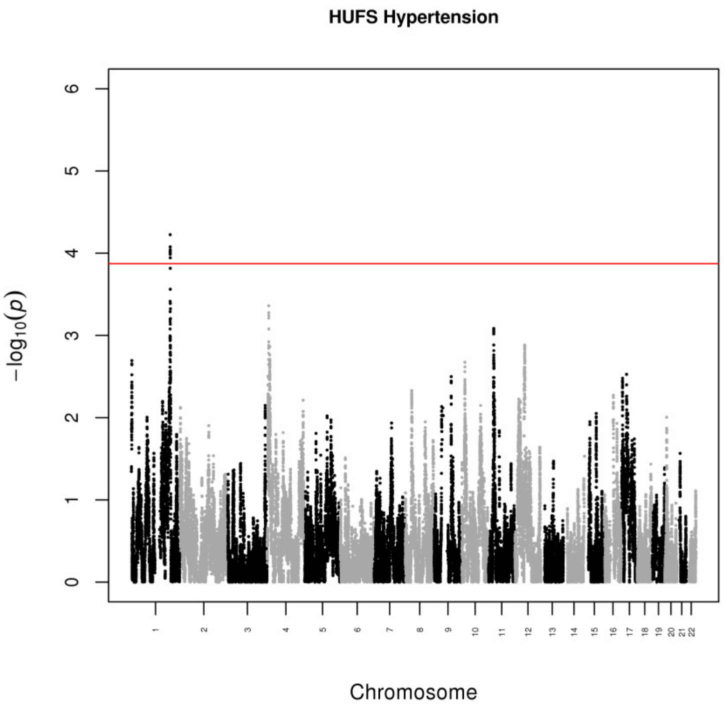
HUFS Hypertension
−log10(p)
0
1
2
3
4
5
6
Chromosome
1
2
3
4
5
6
7
8
9
10
11
12
13
14
15
16
17
18
19
20
21
22

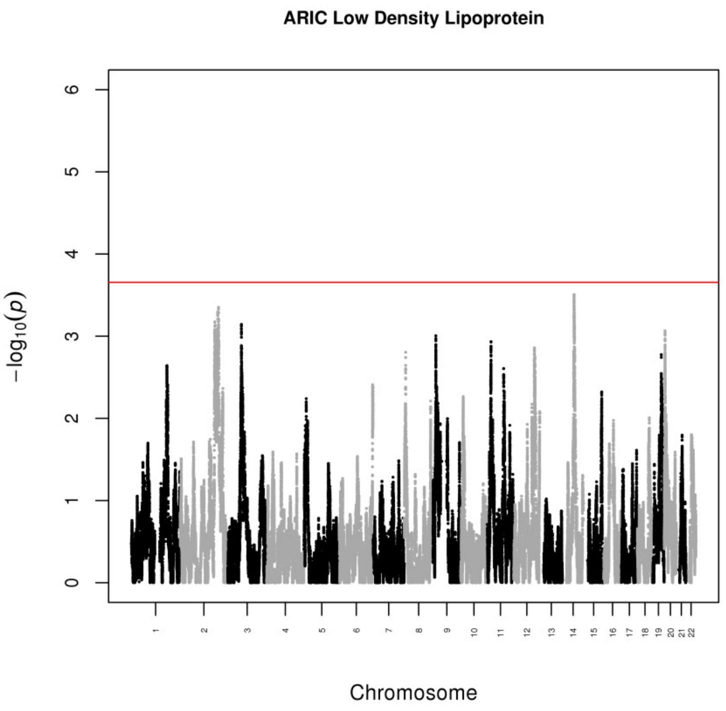
ARIC Low Density Lipoprotein
$-\log_{10}(p)$
0
1
2
3
4
5
6
1
2
3
4
5
6
7
8
9
10
11
12
13
14
15
16
17
18
19
20
21
22
Chromosome

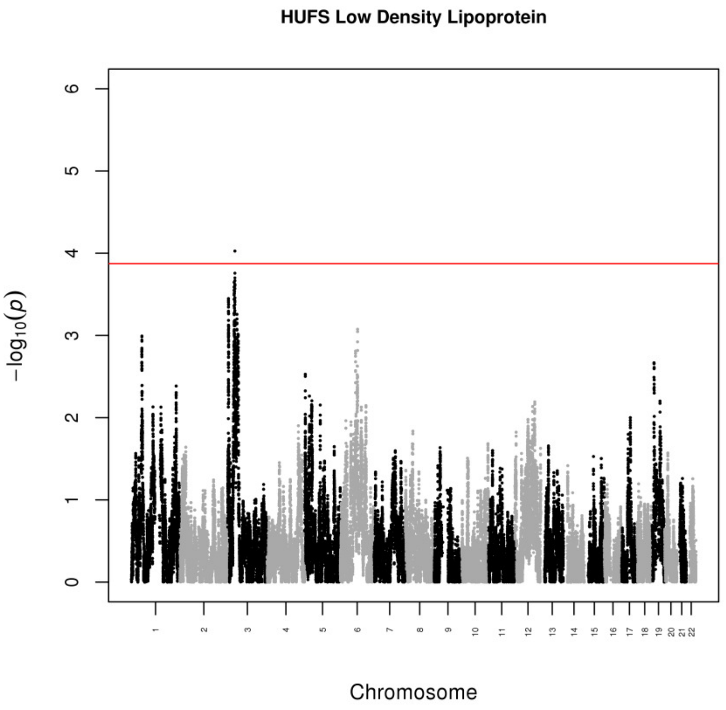
HUFS Low Density Lipoprotein
$-\log_{10}(p)$
0
1
2
3
4
5
6
1
2
3
4
5
6
7
8
9
10
11
12
13
14
15
16
17
18
19
20
21
22
Chromosome

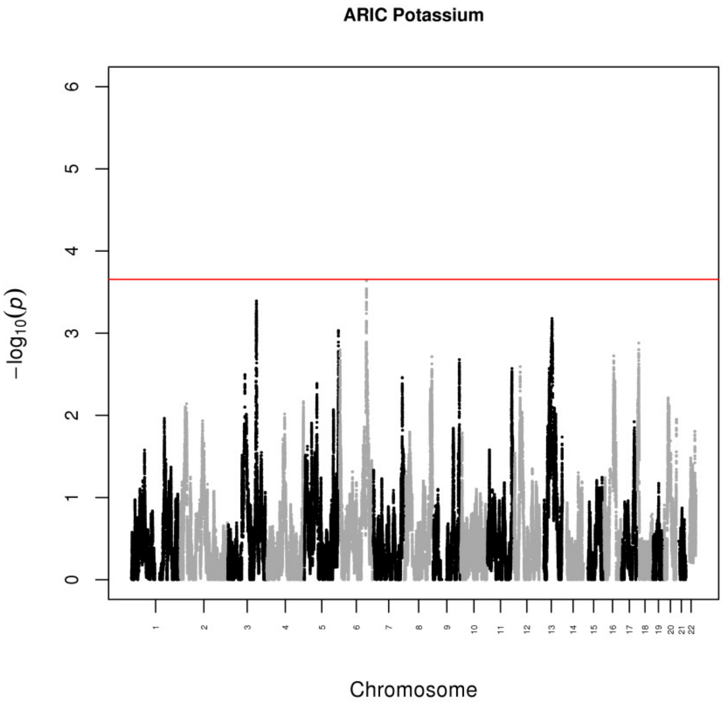
ARIC Potassium
−log10(p)
0
1
2
3
4
5
6
Chromosome
1
2
3
4
5
6
7
8
9
10
11
12
13
14
15
16
17
18
19
20
21
22

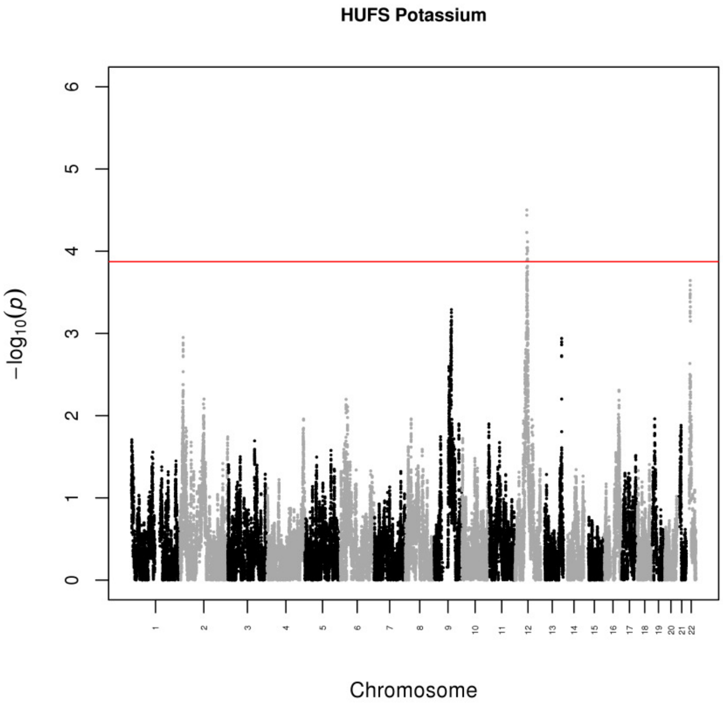
HUFS Potassium
−log10(p)
0
1
2
3
4
5
6
Chromosome
1
2
3
4
5
6
7
8
9
10
11
12
13
14
15
16
17
18
19
20
21
22

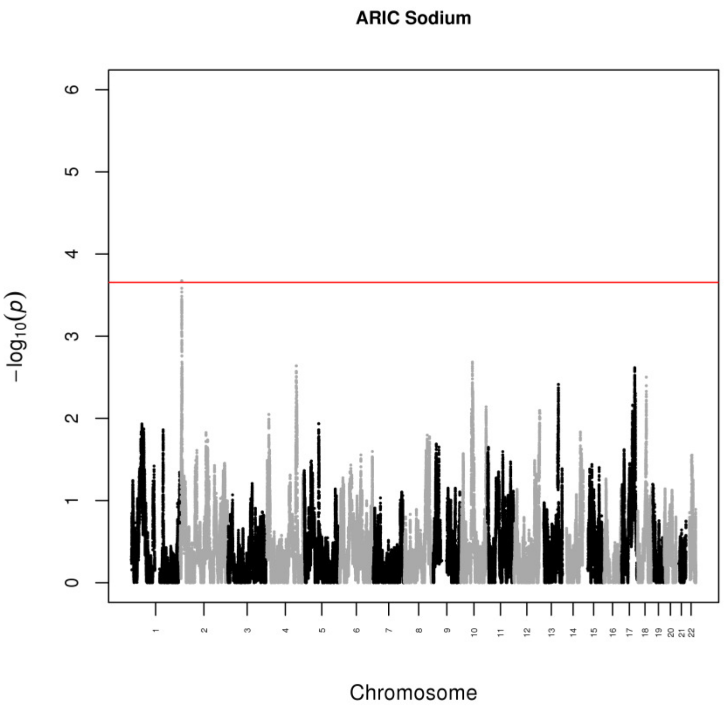
ARIC Sodium
$-\log_{10}(p)$
Chromosome
0
1
2
3
4
5
6
1 2 3 4 5 6 7 8 9 10 11 12 13 14 15 16 17 18 19 20 21 22

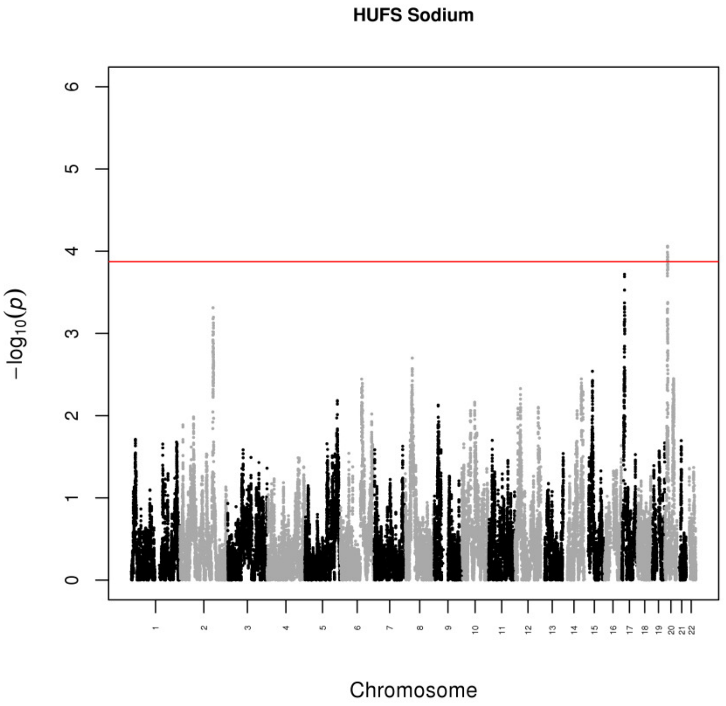
HUFS Sodium
$-\log_{10}(p)$
Chromosome
0
1
2
3
4
5
6
1 2 3 4 5 6 7 8 9 10 11 12 13 14 15 16 17 18 19 20 21 22

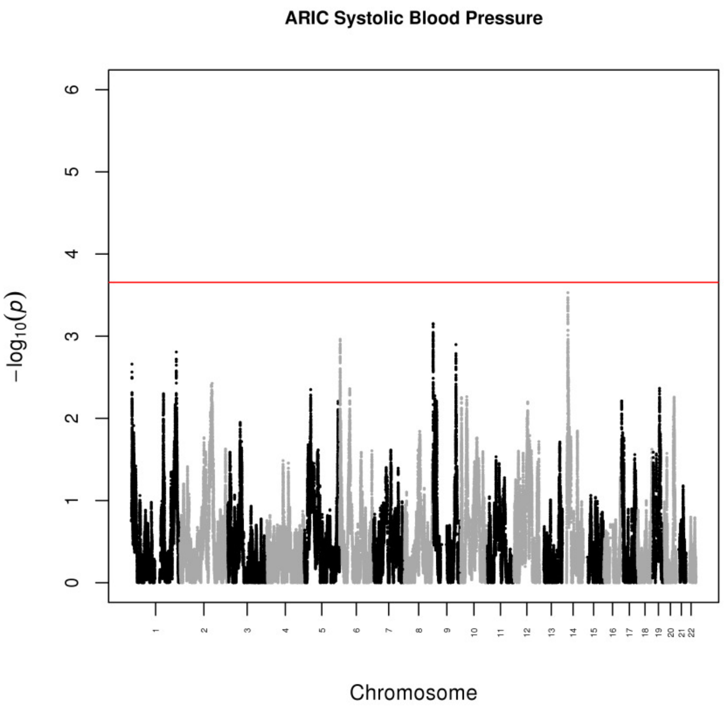
ARIC Systolic Blood Pressure
6
5
4
3
2
1
0
$-\log_{10}(p)$
1
2
3
4
5
6
7
8
9
10
11
12
13
14
15
16
17
18
19
20
21
22
Chromosome

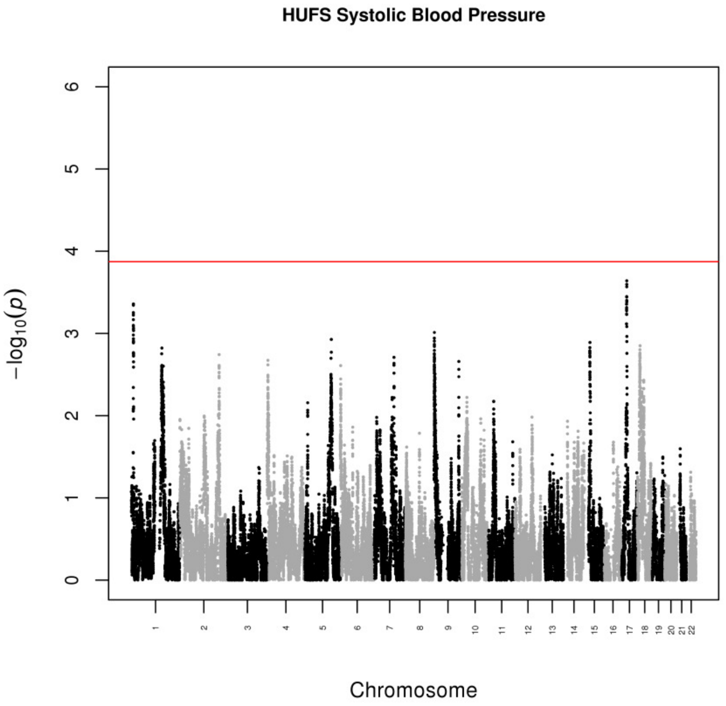
HUFS Systolic Blood Pressure
6
5
4
3
2
1
0
$-\log_{10}(p)$
1
2
3
4
5
6
7
8
9
10
11
12
13
14
15
16
17
18
19
20
21
22
Chromosome

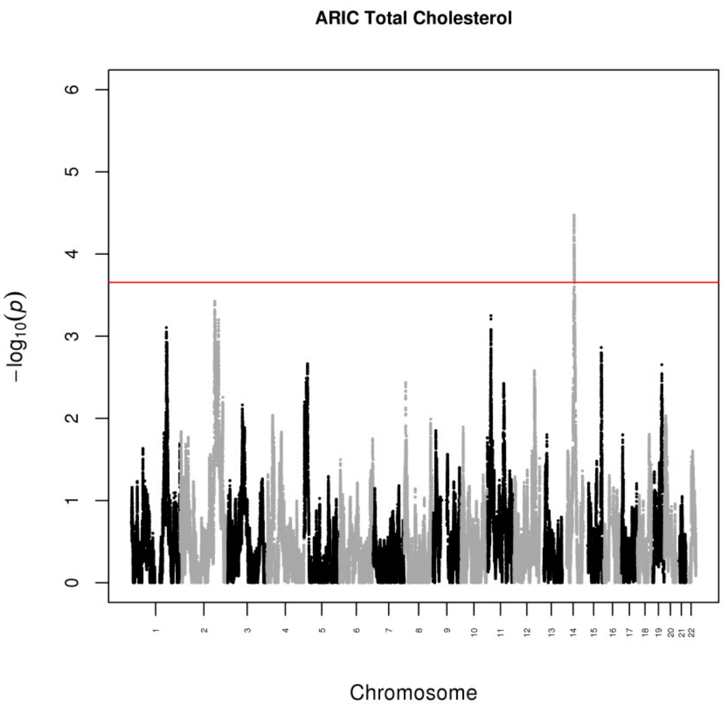
ARIC Total Cholesterol
0
1
2
3
4
5
6
$-\log_{10}(p)$
1
2
3
4
5
6
7
8
9
10
11
12
13
14
15
16
17
18
19
20
21
22
Chromosome

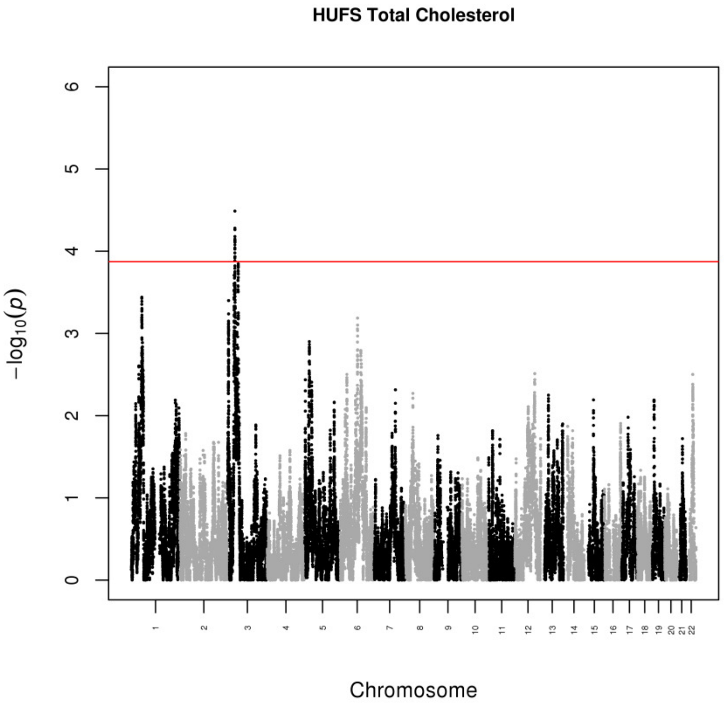
HUFS Total Cholesterol
0
1
2
3
4
5
6
$-\log_{10}(p)$
1
2
3
4
5
6
7
8
9
10
11
12
13
14
15
16
17
18
19
20
21
22
Chromosome

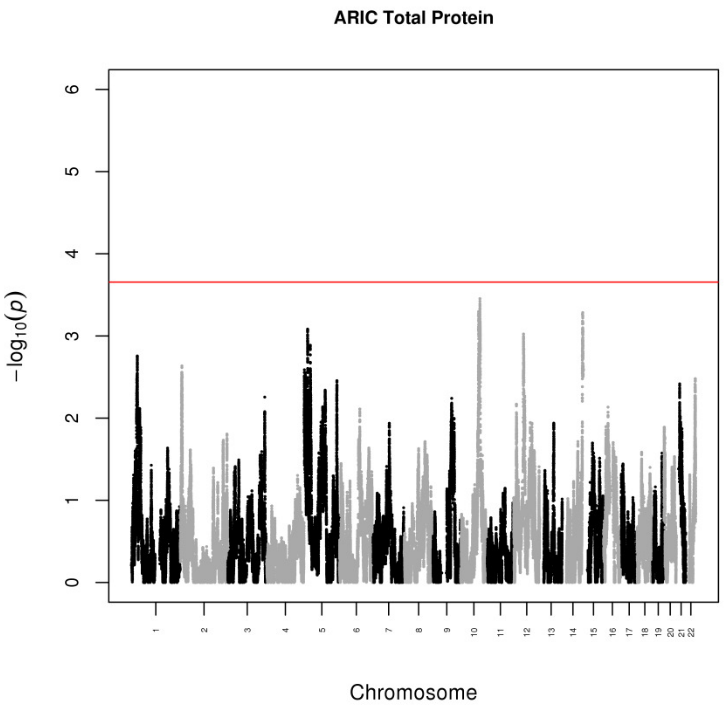
ARIC Total Protein
−log10(p)
0
1
2
3
4
5
6
1
2
3
4
5
6
7
8
9
10
11
12
13
14
15
16
17
18
19
20
21
22
Chromosome

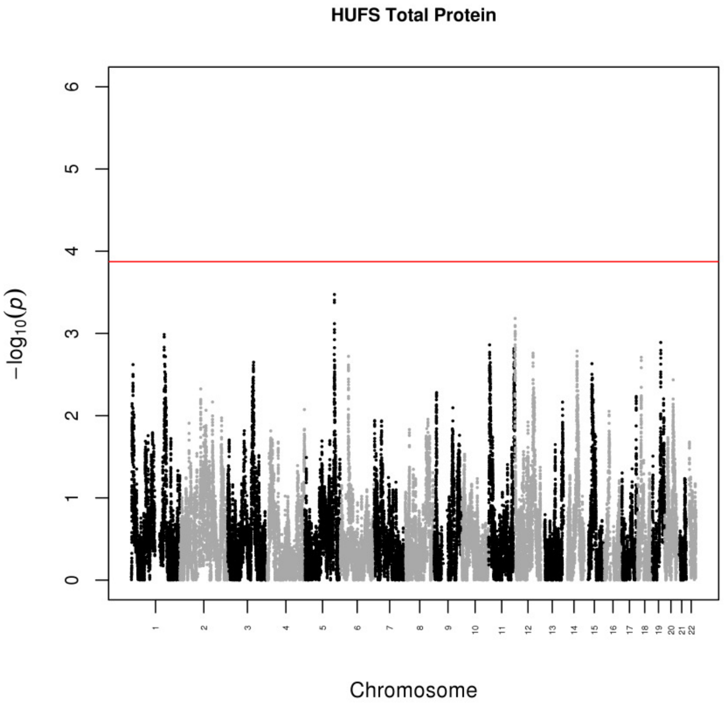
HUFS Total Protein
−log10(p)
0
1
2
3
4
5
6
1
2
3
4
5
6
7
8
9
10
11
12
13
14
15
16
17
18
19
20
21
22
Chromosome

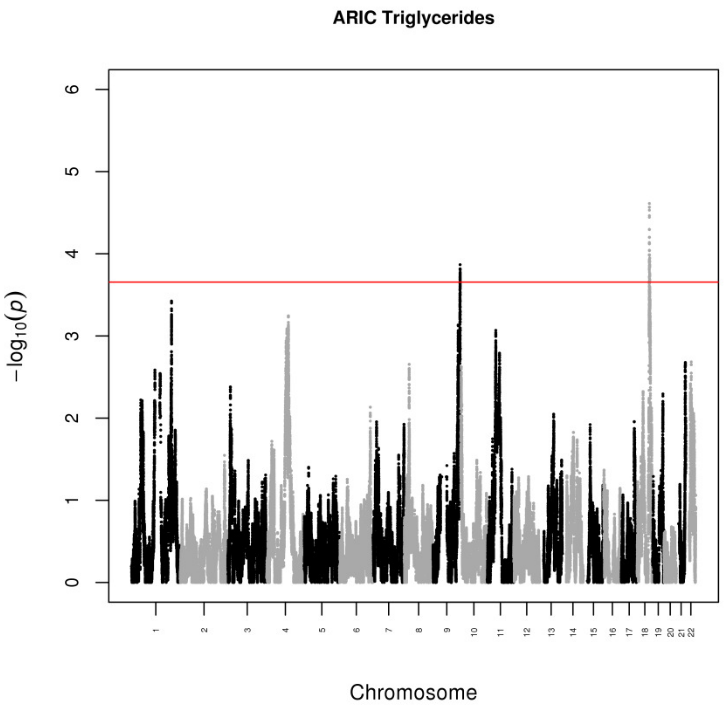
ARIC Triglycerides
−log10(p)
0
1
2
3
4
5
6
Chromosome
1
2
3
4
5
6
7
8
9
10
11
12
13
14
15
16
17
18
19
20
21
22

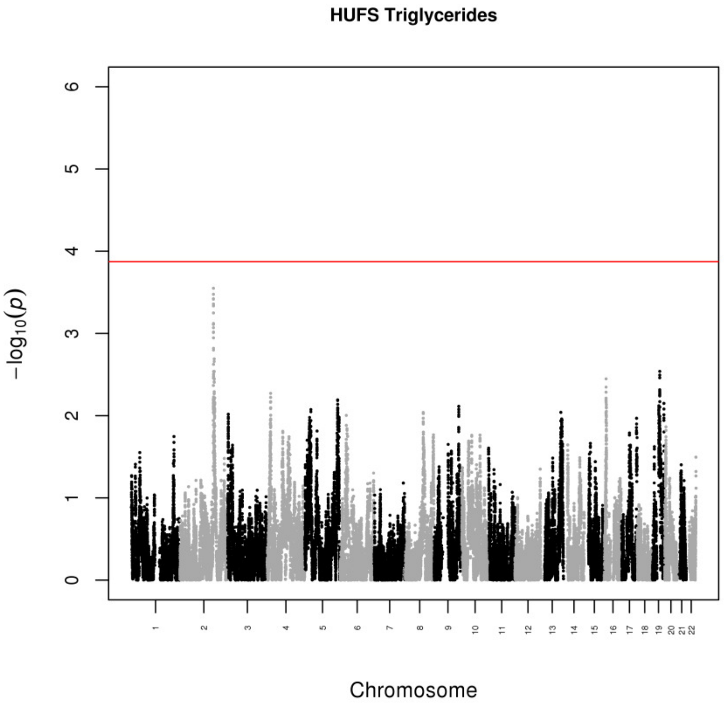
HUFS Triglycerides
−log10(p)
0
1
2
3
4
5
6
Chromosome
1
2
3
4
5
6
7
8
9
10
11
12
13
14
15
16
17
18
19
20
21
22

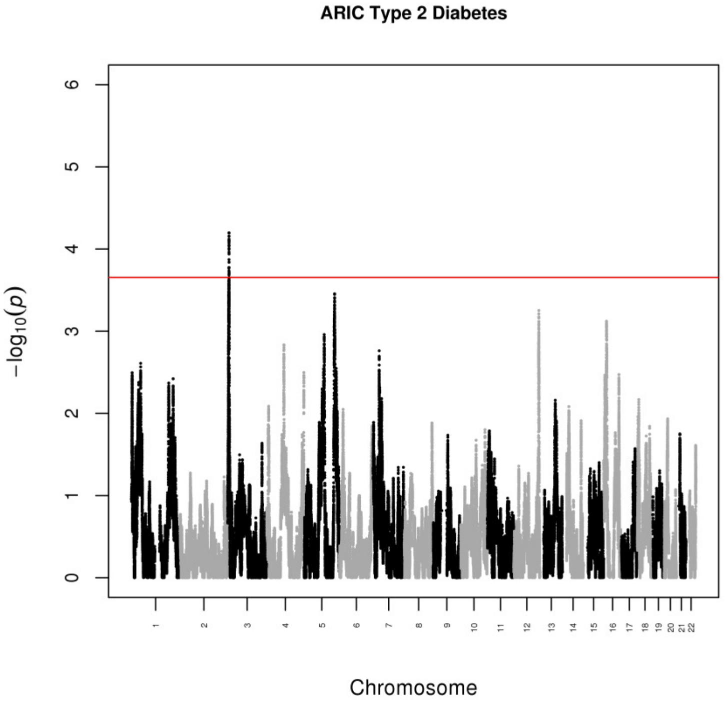
ARIC Type 2 Diabetes
$-\log_{10}(p)$
Chromosome

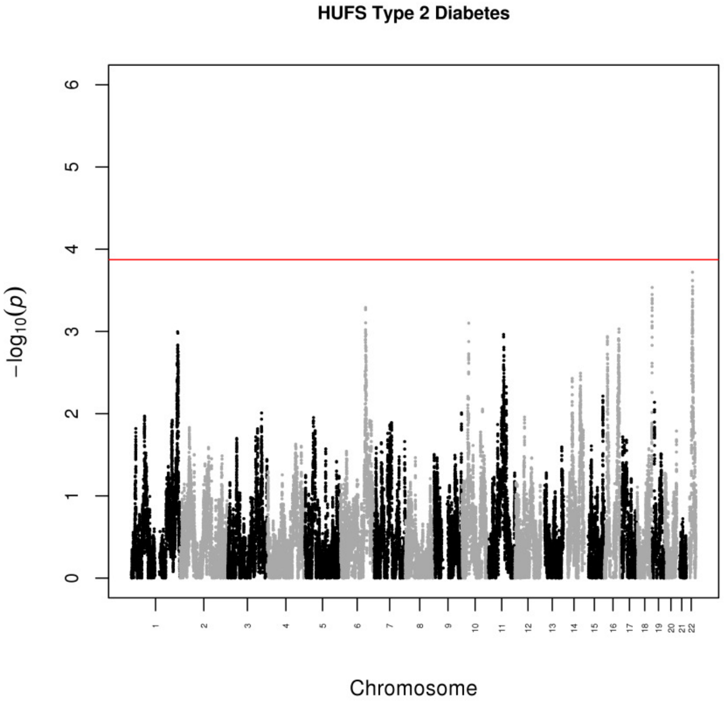
HUFS Type 2 Diabetes
$-\log_{10}(p)$
Chromosome

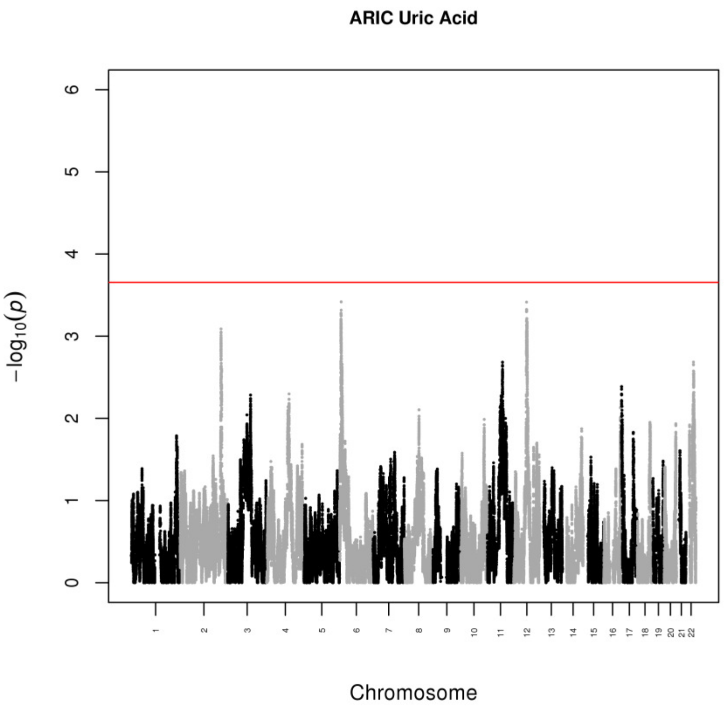
ARIC Uric Acid
6
5
4
3
2
1
0
$-\log_{10}(p)$
1
2
3
4
5
6
7
8
9
10
11
12
13
14
15
16
17
18
19
20
21
22
Chromosome

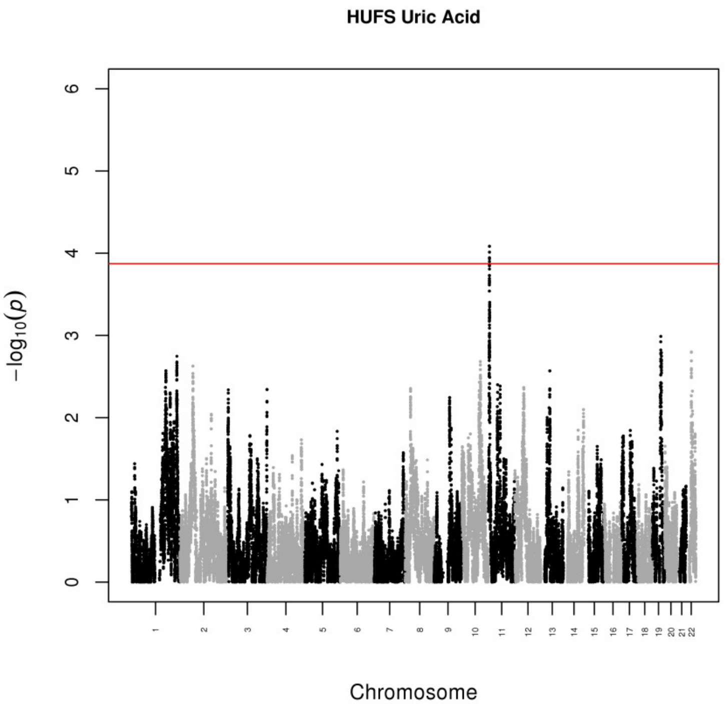
HUFS Uric Acid
6
5
4
3
2
1
0
$-\log_{10}(p)$
1
2
3
4
5
6
7
8
9
10
11
12
13
14
15
16
17
18
19
20
21
22
Chromosome

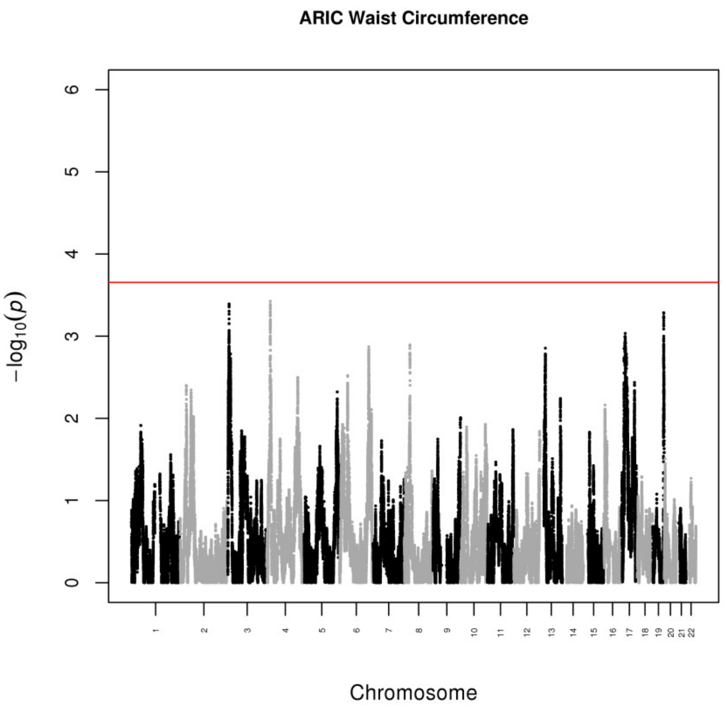
ARIC Waist Circumference
$-\log_{10}(p)$
0
1
2
3
4
5
6
1 2 3 4 5 6 7 8 9 10 11 12 13 14 15 16 17 18 19 20 21 22
Chromosome

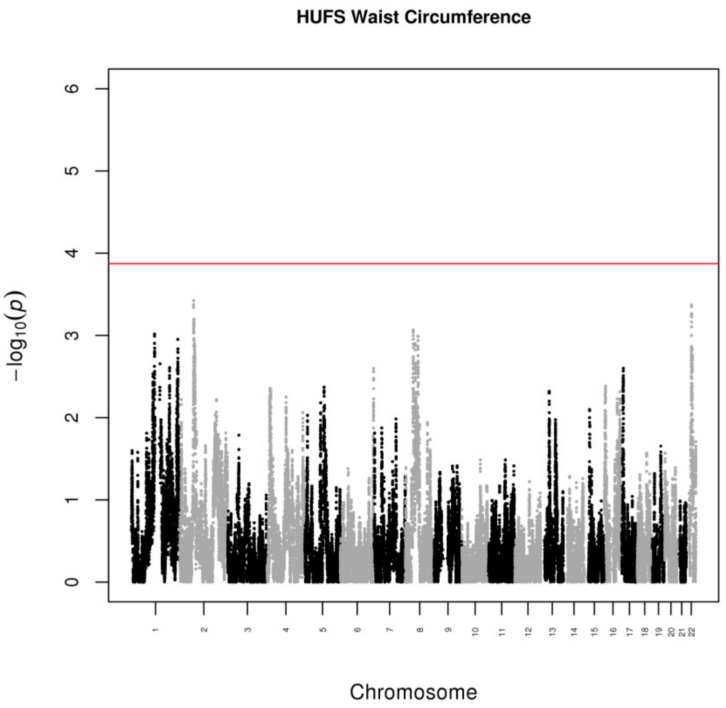
HUFS Waist Circumference
$-\log_{10}(p)$
0
1
2
3
4
5
6
1 2 3 4 5 6 7 8 9 10 11 12 13 14 15 16 17 18 19 20 21 22
Chromosome

ARIC Waist-Hip Ratio

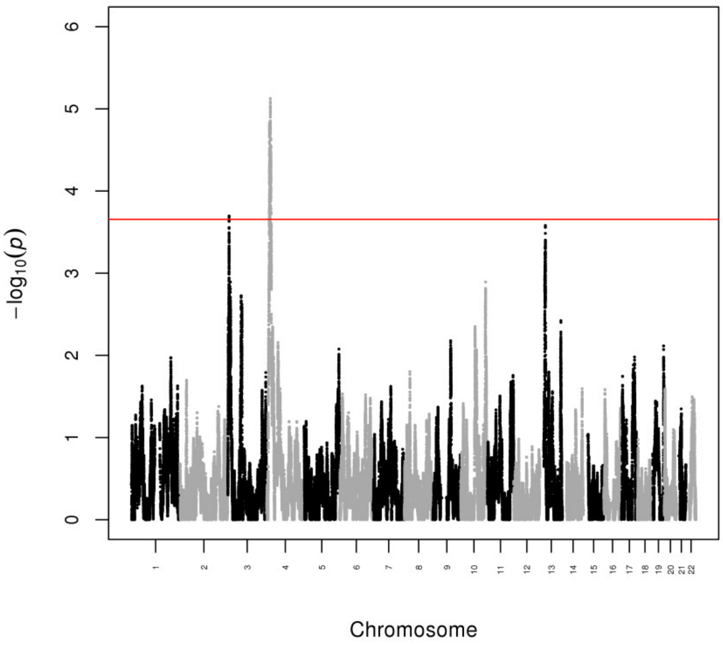

HUFS Waist-Hip Ratio

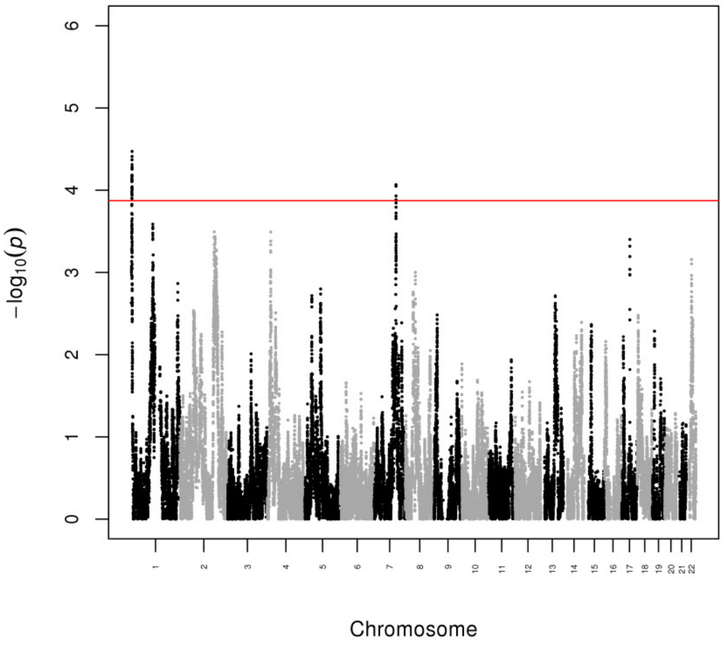

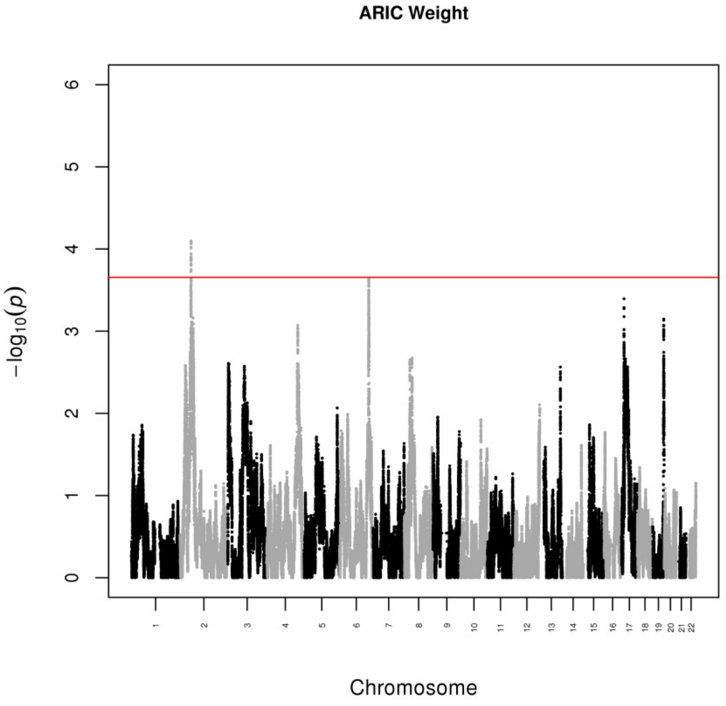

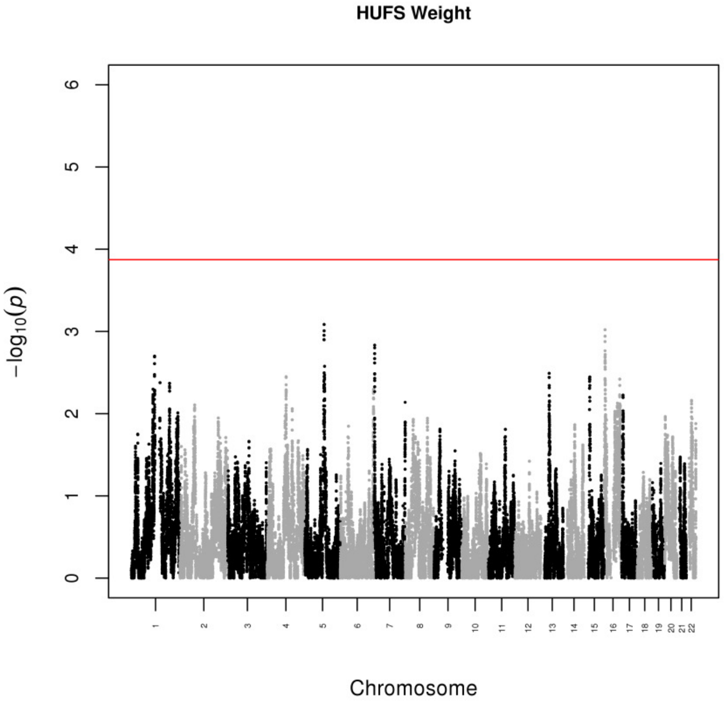

Figure S3. Manhattan plots from admixture mapping for 25 phenotypes in ARIC and HUFS. The red line indicates the genome-wide significance level.
